# Integrating multiscale mathematical modeling and multidimensional data reveals the effects of epigenetic instability on acquired drug resistance in cancer

**DOI:** 10.1371/journal.pcbi.1012815

**Published:** 2025-02-14

**Authors:** Shun Wang, Jinzhi Lei, Xiufen Zou, Suoqin Jin

**Affiliations:** 1 School of Mathematics and Statistics, Wuhan University, Wuhan, China; 2 School of Mathematical Sciences, Center for Applied Mathematics, Tiangong University, Tianjin, China; Sorbonne University, FRANCE

## Abstract

Biological and dynamic mechanisms by which Drug-tolerant persister (DTP) cells contribute to the development of acquired drug resistance have not been fully elucidated. Here, by integrating multidimensional data from drug-treated PC9 cells, we developed a novel multiscale mathematical model from an evolutionary perspective that encompasses epigenetic and cellular population dynamics. By coupling stochastic simulation with quantitative analysis, we identified epigenetic instability as the most prominent kinetic feature related to the emergence of DTP cell subpopulations and the effectiveness of intermittent treatment. Moreover, we revealed the optimal schedule for intermittent treatment, including the optimal area for therapeutic time and drug holidays. By leveraging single-cell RNA-seq data characterizing the drug tolerance of lung cancer, we validated the predictions made by our model and further revealed previously unrecognized biological features of DTP cells, such as cell autophagy and migration, as well as new biomarker genes of therapeutic tolerance. Our work not only provides a paradigm for the integration of multiscale mathematical models with newly emerging genomics data but also improves our understanding of the crucial roles of DTP cells and offers guidance for developing new intermittent treatment strategies against acquired drug resistance in cancer.

## Author summary

Drug-tolerant persister (DTP) cells were recently identified as exhibiting a transient phenotype toward acquired drug resistance, which is one of the main obstacles in the fight against cancer. However, the biological and dynamic mechanisms by which DTP cells contribute to the development of acquired drug resistance in cancer have not been fully elucidated, which hampers the development of more effective anticancer therapies. Currently, multidimensional data of acquired drug resistance in cancer are available, which has great significance for integrating different dimensions of data to systematically understand the development of acquired drug resistance and make dynamic decisions regarding anticancer therapies. Here, we developed a novel multiscale mathematical model and a dynamics-based integrative method for multidimensional data to identify new biomarkers for DTP cells. Furthermore, we proposed the optimal schedule of intermittent treatment using the optimization model and predicted the biomarkers for tolerance of intermittent treatment using scRNA-seq data. Our multiscale modeling framework and computational analytical approach for scRNA-seq data provide a new avenue for predicting the dynamic features of DTP cells leading to the development of acquired drug resistance.

## Introduction

Although significant progress in patient progression has been achieved with new therapies, including immunotherapy, drug resistance remains a major barrier to effective cancer treatment. In addition to genetic events mediating primary resistance, acquired drug resistance, as a nongenetic adaptation mechanism, is believed to play a key role in cancer relapse [1]. A growing body of evidence shows that acquired drug resistance is mediated by a drug-tolerant persister (DTP) cell subpopulation present in tumors, and these cells have been observed in many cancers, such as breast cancer, prostate cancer, and lung cancer [2]. DTP cells are a subpopulation of cancer cells that survive drug treatments, often through reversible adaptations rather than permanen"t resistance mutations [7]. DTP cells have a high survival potential and low proliferation potential, while resistant cells exhibit both high survival and high proliferation potential. Experimental data have shown that simulation of the acute response to various anticancer agents in drug-sensitive cancer cells leads to the emergence of DTP cell subpopulations while the majority of the cell population is instantly killed [3]. After expansion of a single drug-sensitive cancer cell, DTP cells are observed, and their frequency is significantly greater than that observed through mutational mechanisms, indicating epigenetic regulation [4]. The phenotypic state transitions of DTP cells following drug treatment appear to be both frequent and chaotic, generating high heterogeneity. Although several types of DTP cells have been characterized through the expression of ABCB5, CD133, CD271, JARID1B, or aldehyde dehydrogenase, some DTP cell subpopulations have overlapping markers, which poses challenges for tracking their fate [2].

Mathematical modeling is increasingly recognized as an effective tool to interpret the mechanism underlying the progression of carcinoma. By integrating multiple data associated with inflammation and cancer to develop a multiscale computational framework of the long-term evolutionary dynamics leading from inflammation to cancer, cancer risk can be quantified based on the discovery of driver pathways in inflammation-induced tumorigenesis [5]. Several previous studies developed mathematical models for the emergence of DTP cells to uncover their impacts on the evolution of acquired drug resistance. An ODE model of three cell types, namely, drug-sensitive cells, DTP cells and drug-resistant cells, was developed to capture the development of resistance in lung cancer [6]. Using clinical data of patients after treatment, the effect of pre-existing resistance and DTP cell populations on the progression of cancer resistance was previously quantified. Furthermore, a phenotype evolution model has been proposed to explain the transient emergence of drug tolerance [7]. In this model, the proliferation and survival potentials serve as phenotypic information to characterize three distinct subpopulations: parental cancer cells, DTP cells, and drug-tolerant expanded persister cells. This work revealed that nongenetic factors during phenotype evolution drive parental cells to transition into DTP cells. However, quantifying the survival and proliferative potentials in biological experiments is challenging. In a recent study, the “chance to persist” (CTP) is found as a highly stable inherited trait of drug persistance and enriched when exposed to cytotoxic drugs [8]. This trait is an altered epigenetic state that is heritable and regulates the ability of cell proliferation and cell apoptosis [9]. In biological experiments, CTP is determined by calculating the quotient of the number of cells after 7–9 days of treatment divided by the number of cells before the treatment is initiated (day 1) [8]. Motivated by this experimental work, a single-cell probability density model was proposed to explain the emergence of cancer cell persistence [9]. This model provides a quantitative approach for drug-exposed populations of cancer cells with the aim of understanding recent experimental data regarding the inheritance of DTP cell subpopulations [9]. However, these studies do not offer a systematic and dynamic understanding of the evolution of acquired drug resistance in cancer across multiple scales, including population dynamics at the macroscale and epigenetic dynamics at the microscale. Notably, they did not utilize biological data to validate the model’s predictions or identify specific biomarkers to predict the evolution of acquired resistance—both of which are crucial for enabling precise drug interventions to prevent cancer recurrence in clinical practice.

**Fig 1:**
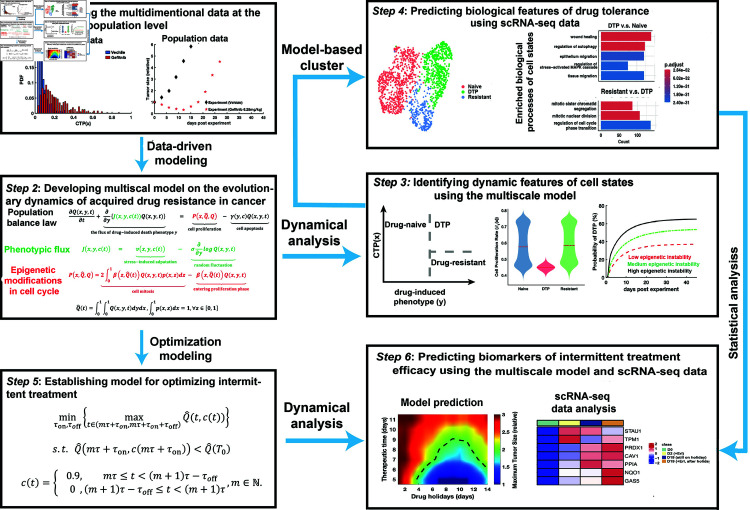
A framework of dynamics-based multidimensional data integration.

To address this limitation, we developed a multiscale computational model of epigenetic population evolution with the aim of revealing the underlying mechanisms of acquired drug resistance. By integrating multidimensional data from the PC9 cell line treated with gefitinib, our model reconstructs the epigenetic population dynamics of heterogeneous cell populations subjected to gefitinib treatment. Based on this computational model, we proposed a mathematical description of epigenetic inheritance for three types of cells—drug-naïve cells, DTP cells, and drug-resistant cells—and then identified the distinctive features of DTP cell subpopulations. Based on these biological features predicted by the model, we clustered a single-cell RNA-seq (scRNA-seq) data on drug tolerance and identified three cell types described in our model, allowing us to further identify potential biomarkers for the DTP cell subpopulations. Moreover, we established an optimization model to provide a quantitative schedule for intermittent treatment for acquired drug resistance. By analyzing the scRNA-seq data with the drug holidays, we further identified the differentially expressed genes associated with epigenetic instability associated with tolerance to drug treatment after drug holidays. Our study not only sheds new light for understanding the development of acquired drug resistance but also provides a potential guide for intermittent therapy against acquired drug resistance in lung cancer. The entire workflow of this study is depicted in Fig 1.

## Materials and methods

### Collection of data from the published literature

**Dataset 1: Distribution of the epigenetic CTP state of PC9 cells treated with gefitinib for seven days.** The data were retrieved from a reference that included 544 clones that were grown for 12–20 days and then treated and 392 clones that were generated after 9 days of treatment with gefitinib [8].

**Dataset 2: Kinetic data of the tumor volume of mice transplanted with lung adenocarcinoma cell lines (PC9)**. The dataset included data on the tumor volume dynamics of the gefitinib and vehicle group [10].

**Dataset 3: scRNA-seq data of drug tolerance in lung cancer.** The scRNA-seq data of drug tolerance in lung cancer included three experiments: (1) The original PC9 cells were treated with 2 *μM* erlotinib for the specified days (D1, D2, D4, D9, and D11); (2) The original PC9 cells were treated with a different drug at 2 *μM* after three days; (3) Intermittent therapy for PC9 cells treated with 2 *μM* erlotinib. The specific regimen for intermittent treatment is 11 days of treatment with 2 *μM* erlotinib, followed by an 8-day break, and then two additional days of treatment with 2 *μM* erlotinib [11].

Dataset 1 was used to estimate the parameters for the inheritance function at the epigenetic level. Dataset 2 was utilized to estimate the parameters related to cell behaviors including cell proliferation and apoptosis. The procedures used for Dataset 1 and Dataset 2 are displayed in the Supplementary Section titled Parameter estimation in S1 Text. Dataset 3 was used to validate our theoretical results and predict the potential biomarkers of drug tolerance.

### Multiscale model describing the evolutionary dynamics of acquired drug resistance

**Fig 2:**
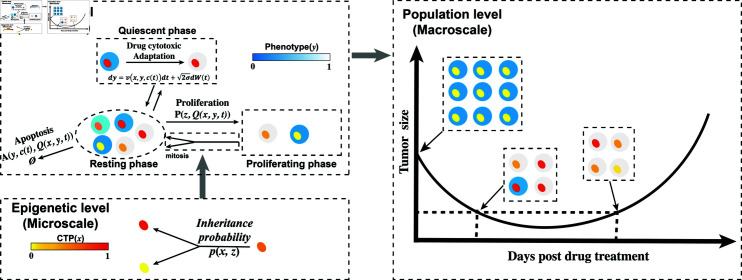
Multiscale model illustration. At the population level, the combination of population balance laws governs the dynamics of phenotypes related to cellular apoptosis induced by drug cytotoxicity [9,12] and epigenetic inheritance during the process of cell proliferation [13]. During the resting phase, cells reversibly migrate to the quiescent phase [14] and vice versa under the stress induced by drug cytotoxicity. At the epigenetic level, the epigenetic information from a mother cell is inherited by the two daughter cells with the probability *p* ( *x* , *z* ) .

We described the epigenetic evolution of a cancer cell population exposed to a cytotoxic drug whose concentration at time *t* is *c*(*t*) using population balance analysis (PBA) [12] and the epigenetic dynamics during the cell proliferation process [13] (Fig 2).

In general, the infusion of the cytotoxic drug was set to a constant (i.e., *c* ( ⋅ ) : = *c* > 0). We characterized the state of cancer cells according to their epigenetic state and phenotype. Based on a previous study [8], the epigenetic state was quantified by CTP, which was denoted as the continuous variable *x* (*CTP* ≡ *x*) ranging from 0 to 1  ( 0 < *x* < 1 ) . The phenotype of cancer cells is a projection of the phenotypic degrees of freedom onto the one-dimensional axis related to drug-induced death (*y*) [9], the range of which was 0 to 1 (0 < *y* < 1).

#### Phenotypic dynamics.

Now, we denoted *Q* ( *x* , *y* , *t* )  as the number of cells with CTP (*x*) and phenotype (*y*) at time *t* in the resting phase. Because of the stress induced by drug cytotoxicity, resting-phase cells migrate to the quiescent phase, and their phenotype changes to adapt to the stress. Quiescent cells are not actively proliferating, whereas resting cells are preparing to enter the proliferation phase [14]. In our mathematical model, the phenotype variable (*y*) of cells in the quiescent phase is affected by their epigenetic variable (*x*), while the phenotype variable (*y*) of cells in the resting phase remains unchanged. Due to the low proliferative rate of DTP cells, they are predominantly quiescent. For simplicity, we assumed that if the cells remain in the resting phase, their phenotype (*y*) can be represented by the following word equation:


$$\begin{array}{c} \begin{array}{ccc} \text{Change in the number of cells with (}x\text{) and (}y\text{) at time }t\;+ & \text{the flux of phenotype (}y\text{)} & \\ = & \text{cell proliferation}-\text{cell apoptosis.} \end{array} \end{array}$$ (1)

The mathematical formulation for this equation is as follows:


$$\begin{array}{c} \begin{array}{ccc} \frac{\partial Q(x,y,t)}{\partial t}+\underset{\text{the flux of phenotype (}y\text{) }}{\underbrace{\frac{\partial }{\partial y}\{J(x,y,c(t))Q(x,y,t)\}}} & = & \underset{\text{cell proliferation}}{\underbrace{P(x,\hat{Q},Q)}}-\underset{\text{cell apoptosis}}{\underbrace{A(y,c,Q)}}. \end{array} \end{array}$$ (2)

where $\hat{Q}$ is the total cell number ($\hat{Q}(t)=\int_{0}^{1}\int_{0}^{1}Q(x,y,t)dydx$), and the flux of phenotype *J* ( *x* , *y* , *t* )  can be decomposed into stress-induced adaptation to cell survival and random fluctuation in *y*, as shown in Eq (3).


$$\begin{array}{c} \begin{array}{ccc} J(x,y,c(t)) & = & \underset{\text{stress-induced adpatation}}{\underbrace{v(x,y,c(t))}}-\underset{\text{random fluctuation}}{\underbrace{\sigma \frac{\partial }{\partial y}logQ(x,y,t)}}. \end{array} \end{array}$$ (3)

In addition, the boundary condition of the flux *J* ( *x* , *y* , *t* )  is a reflecting barrier (*J* ( *x* , *y* , *t* ) ⋅ =0, is the normal vector at the boundary). Under the pressure of drug treatment, cells release signaling molecules to regulate the apoptosis pathway induced by cytotoxic drugs, which is considered an adaptation of the cell phenotype to drug toxicity [7]. *v* ( *x* , *y* , *c* ( *t* ) ) , which denotes the speed of the stress-induced adaptation of the cell survival level, represents the phenotypic flux characterized as a phenotypic switch under the pressure of drug cytotoxicity [7,9]. *σ* represents the magnitude of the random fluctuation in phenotype (*y*) [9]. The function *v* ( *x* , *y* , *c* ( *t* ) )  considers the impact of stress-induced adaptation on the level of cell survival. The dependence of *v* on the concentration of the cytotoxic drug *c*(*t*) reflects that the rate of stress-induced adaptation depends on the cytotoxic drug concentration c(t) in the cell’s local environment, as shown in Eq (4).


$$\begin{array}{ccc} v\left(x,y,c(t)\right)=\{\begin{array}{ccc} v_{0}xc(t)(y_{0}(c)-y),\;y_{0}(c)=0.8 & & c>0\\ v_{0}x(y_{0}(c)-y),\;y_{0}(c)=0.2 & & c=0 \end{array}. & & \end{array}$$ (4)

Herein, $y_{0}(c)$ was the equilibrium point of cell survival under the different environments, and $v_{0}$ represents the cell stress-induced adaptation. Cell apoptosis in the resting phase includes normal cell death and drug-induced cell apoptosis that is dependent on the phenotype (*y*). Therefore, the formula for cell apoptosis is given as follows:


$$\begin{array}{c} \begin{array}{ccc} A(y,c,Q) & =\gamma (y,c)Q(x,y,t) & \\ & =\underset{\text{natural death}}{\underbrace{\gamma_{0}Q(x,y,t)}}+\underset{\text{death induced by drug}}{\underbrace{\gamma_{1}c(t)H(y^{*}-y)Q(x,y,t)}}. \end{array} \end{array}$$ (5)

where *H*(*y*) is the Heaviside function and the threshold value of $y^{*}$ was set to 0.5. Similar to previous work [9] , the Heaviside function is used to represent the drug-induced cell apoptosis rate associated with phenotype *y*. The parameters $\gamma_{0}$ and $\gamma_{1}$ represent the natural death rate and drug-induced death rate, respectively. *c*(*t*) is the coefficient of drug cytotoxicity, where *c* ( *t* ) = 1 is associated with the maximum tolerance dose and *c* ( *t* ) = 0 is associated with the zero dose (*c* ( *t* ) ∈ [ 0 , 1 ] ).

#### Epigenetic dynamics.

Epigenetic dynamics is characterized by cell proliferation process (Fig 2). The resting-phase cells can either re-enter the proliferative phase at a rate *β* that involves negative feedback from cell populations. The epigenetic dynamic model is used to simulate the redistribution of the epigenetic state following cell division and represents the stochastic partitioning of molecules carrying inheritable information when a mother cell divides into two daughter cells during the M phase of mitosis [13]. We established the epigenetic dynamics using the following word equation:


$$\begin{array}{c} \begin{array}{ccc} \text{Cell proliferation with }x\text{ and }y & =\text{two daughter cells with }x\text{ and }y\; & \\ & \text{ from all mother cells with }y\\ & -\text{cells with }x\text{ and }y\text{ entering the proliferating phase.} \end{array} \end{array}$$ (6)

The mathematical formulation was written as follows:


$$\begin{array}{c} \begin{array}{ccc} P(x,\hat{Q},Q) & = & \underset{\text{cell mitosis}}{\underbrace{2\int_{0}^{1}\beta (z,\hat{Q}(t))Q(z,y,t)p(x,z)dz}}-\underset{\text{entering proliferation phase}}{\underbrace{\beta (x,\hat{Q}(t))Q(x,y,t)}}. \end{array} \end{array}$$ (7)

Here, $\beta (x,\hat{Q}(t))$represents the rate of cells entering the proliferation phase from the resting phase (More details shown in Supplementary Section 3 in S1 Text). This function is regulated by the CTP state (*x*) and the total size of the cell population ($\hat{Q}$), which is mathematically expressed [13] as follows:


$$\begin{array}{c} \begin{array}{c} \beta (x,\hat{Q}(t))=\beta_{1}(x)(1-\frac{\hat{Q}}{K}),\;\beta_{1}(x)=\beta_{0}+\beta_{10}\frac{a_{1}x+{\left(a_{2}x\right)}^{6}}{1+{\left(a_{3}x\right)}^{6}}. \end{array} \end{array}$$ (8)

where $\beta_{1}(x)$ is the rate of cell proliferation regulated by CTP, and the parameters $\beta_{0},\beta_{10},a_{1},a_{2}$, and $a_{3}$ are constants [13]. We choose this form of $\beta_{1}(x)$ based on the relevant work on heterogeneous cell proliferation in a previous study [13]. Additionally, we found that this form was able to capture the relationship between $\beta_{1}(x)$ and the CTP level x observed from the CTP experiment in which high CTP levels correlate with a low net growth rate [9]. The function *p* ( *x* , *z* )  represents the inheritance probability of CTP (*x*) as *p* ( *x* , *z* ) =  P(CTP of daughter cell= CTP of mother cell= as follows:


$$\begin{array}{c} \begin{array}{c} \int_{0}^{1}p(x,z)dx=1,\forall z\in [0,1]. \end{array} \end{array}$$ (9)

By integrating the Eqs (2)–(8), the mathematical formulation of the multiscale model can be given by the following:


$$\begin{array}{c} \begin{array}{ccc} \frac{\partial Q(x,y,t)}{\partial t}= & -\frac{\partial }{\partial y}\{v(x,y,c(t))Q(x,y,t)\}+\sigma \frac{\partial^{2}}{\partial y^{2}}Q(x,y,t)-\gamma (y,c)Q(x,y,t) & \\ & +2\int_{0}^{1}\beta (z,\hat{Q}(t))Q(z,y,t)p(x,z)dz-\beta (x,\hat{Q}(t))Q(x,y,t). \end{array} \end{array}$$ (10)

where it has $\hat{Q}(t)=\int_{0}^{1}\int_{0}^{1}Q(x,y,t)dydx$, and $\int_{0}^{1}p(x,z)dx=1,\forall z\in [0,1]$, and *p* ( *x* , *z* )  follows a Beta distribution (for more details, see the section on the Inheritance function).

### Single-cell epigenetic dynamics of acquired drug resistance

The multiscale model describes the heterogeneous cell populations that integrate epigenetic dynamics at the microscale and cell population dynamics at the macroscale. Based on the Eq (10), we further proposed a mathematical formulation for the regulation of the cell fate by epigenetic state at the microscale. First, we denoted the probability density of a single cell with CTP (*x*) and phenotype (*y*) as


$$\begin{array}{c} \begin{array}{ccc} f(x,y,t) & = & \frac{Q(x,y,t)}{\hat{Q}(t)},\;\int_{0}^{1}\int_{0}^{1}f(x,y,t)dxdy=1. \end{array} \end{array}$$ (11)

Using the chain law, we can easily obtain


$$\begin{array}{c} \begin{array}{ccc} \frac{\partial f(x,y,t)}{\partial t} & = & \frac{1}{\hat{Q}(t)}(\frac{\partial Q(x,y,t)}{\partial t}-f(x,y,t)\frac{d\hat{Q}(t)}{dt}). \end{array} \end{array}$$ (12)

Note that the flux *J* ( *x* , *y* , *c* ( *t* ) )  is a reflecting barrier, which was obtained by Green’s law as


$$\begin{array}{c} \begin{array}{ccc} \int_{0}^{1}\int_{0}^{1}\frac{\partial }{\partial y}\{J(x,y,c(t))Q(x,y,t)\}dxdy & = & \int_{\partial \Omega }Q(x,y,t)J(x,y,c(t))\cdot \text{n}ds=0. \end{array} \end{array}$$ (13)

Now, the derivative of the total cell number with respect to time *t* is given as:


$$\begin{array}{c} \begin{array}{ccc} \frac{d\hat{Q}}{dt} & = & \int_{0}^{1}\int_{0}^{1}(\beta (x,\hat{Q}(t))-\gamma (y,c))Q(x,y,t)dxdy. \end{array} \end{array}$$ (14)

By integrating Eqs (12) and (14), the derivative of the probability density of the cell with CTP (*x*) and phenotype (*y*) with respect to time *t* is given as follows:


$$\begin{array}{c} \begin{array}{ccc} \frac{\partial f(x,y,t)}{\partial t}= & -\frac{\partial }{\partial y}\{v(x,y,c(t))f(x,y,t)\}+\sigma \frac{\partial^{2}}{\partial y^{2}}f(x,y,t)-\gamma (y,c)f(x,y,t) & \\ & +2\int_{0}^{1}\beta (z,\hat{Q}(t))f(z,y,t)p(x,z)dz-\beta (x,\hat{Q}(t))f(x,y,t)\\ & -f(x,y,t)\int_{0}^{1}\int_{0}^{1}(\beta (x,\hat{Q}(t))-\gamma (y,c))f(x,y,t)dxdy. \end{array} \end{array}$$ (15)

Next, we proposed a conditional probability for phenotype (*y*) denoted as *h* ( *y* , *t* ; *x* )  and a marginal probability of CTP (*x*) denoted as *g* ( *x* , *t* )  *f* ( *x* , *y* , *t* ) = *h* ( *y* , *t* ; *x* ) *g* ( *x* , *t* ) . Using the chain law, we easily obtain


$$\begin{array}{c} \begin{array}{ccc} \frac{\partial f(x,y,t)}{\partial t} & = & g(x,t)\frac{\partial h(y,t;x)}{\partial t}+h(y,t;x)\frac{\partial g(x,t)}{\partial t}. \end{array} \end{array}$$ (16)

We assumed that cell-cell interactions are ignored in the single-cell case ($\beta (x,\hat{Q}(t))\approx \beta_{1}(x)$), and integrated Eqs (15) and (16) as:


$$\begin{array}{c} \begin{array}{ccc} \frac{\partial h(y,t;x)}{\partial t}= & -\frac{\partial }{\partial y}\{v(x,y,c(t))h(y,t;x)\}+\sigma \frac{\partial^{2}}{\partial y^{2}}h(y,t;x) & \\ & -h(y,t;x)(\gamma (y,c)-\int_{0}^{1}\int_{0}^{1}\gamma (y,c)h(y,t;x)g(x,t)dxdy), \end{array} \end{array}$$ (17)

$$\begin{array}{c} \begin{array}{cc} \frac{\partial g(x,t)}{\partial t} & =2\int_{0}^{1}\beta_{1}(z)g(z,t)p(x,z)dz-g(x,t)(\beta_{1}(x)+\int_{0}^{1}\beta_{1}(z)g(z,t)dz). \end{array} \end{array}$$ (18)

### Inheritance function

The inheritance function *p* ( *x* , *z* )  quantifies the process of cell division, during which daughter cells inherit the CTP value *x* from mother cells with the CTP value *z* through complex regulatory mechanisms that are not fully understood. Based on the previous study [13], we define the inheritance function using the Beta distribution:


$$\begin{array}{c} \begin{array}{c} p(x,z)=\frac{x^{a(z)-1}{\left(1-x\right)}^{b(z)-1}}{B(a(z),b(z))},\;B(a(z),b(z))=\frac{\Gamma (a(z))\Gamma (b(z))}{\Gamma (a(z)+b(z))}. \end{array} \end{array}$$ (19)

where *Γ* ( ⋅ )  represents the gamma function. Herein, the transition function of the beta function was defined by two shape parameters, *a*(*z*) and *b*(*z*), and these parameters are dependent on the mother cell with CTP value *z*. Based on previous work [13], given a mother cell with CTP value *z*, we obtained two functions *ϕ* ( *z* )  and *η* ( *z* )  such that the conditional expectation and variance of the daughter cells with CTP value *x* can be written as


$$\begin{array}{c} \begin{array}{c} E(x|z)=\phi (z),\;Var(x|z)=\frac{1}{1+\eta (z)}\phi (z)(1-\phi (z)). \end{array} \end{array}$$ (20)

It is easy to obtain 0 < *ϕ* ( *z* ) < 1, *η* ( *z* ) > 0. In this case, from Eqs (19) and (20), the shape parameters can be given by *ϕ* ( *z* )  and *η* ( *z* )  as:


$$\begin{array}{c} \begin{array}{c} a(z)=\eta (z)\phi (z),\;b(z)=\eta (z)(1-\phi (z)). \end{array} \end{array}$$ (21)

Furthermore, we constructed the CTP transition function *p* ( *x* , *z* )  with functions *ϕ* ( *z* )  and *η* ( *z* )  defined as follows:


$$\begin{array}{c} \begin{array}{c} \phi (z)=\phi_{0}+\phi_{1}\frac{{\left(\alpha z\right)}^{n}}{1+{\left(\alpha z\right)}^{n}},\;\eta (z)=\eta_{0}>0. \end{array} \end{array}$$ (22)

where *ϕ* ( *z* )  is a boundary function ($\left(\phi (z)\in (\phi_{0},\phi_{0}+\phi_{1})\right)$) and *η* ( *z* )  is constant [13]. Herein, the noise fluctuation of the altered CTP state was dominated by $\eta_{0}$, because the conditional variance of *x* was controlled as follows:


$$\begin{array}{c} \begin{array}{c} Var(x|z)=\frac{1}{1+\eta (z)}\phi (z)(1-\phi (z))\leq \frac{1}{4}\frac{1}{1+\eta (z)}<\frac{1}{4\eta_{0}}. \end{array} \end{array}$$ (23)

The parameter *α* represents the degree of selective pressure favoring a high CTP state, as shown in Fig A in S1 Text. The parameters $\phi_{0}$, $\phi_{1}$ and n are constant. The experiments[8] have confirmed that CTP is a heritable trait. In the control group, the CTP distribution of cells not induced by cytotoxic drugs is concentrated at a low level; however, after seven days of drug induction, the peak of the CTP distribution shifts to a higher level. Based on this biological experimental fact, we proposed the model hypothesis that cell CTP shift to a higher level when exposed to a cytotoxic drug environment. This mathematical function is expressed as


$$\begin{array}{c} \begin{array}{c} \alpha =\alpha_{0}+kc(t). \end{array} \end{array}$$ (24)

Herein, the coefficient *k* is positive. The values for all parameters are displayed in Table A in S1 Text, and the procedures for parameter estimation are shown in the Supplementary Section titled Parameter estimation in S1 Text.

**Quantification of epigenetic instability**. By referring to the relevant quantitative studies [7,15] on the epigenetic regulation of the evolution of acquired drug resistance in cancer, we utilized epigenetic noise ($1∕\eta_{0}$) to quantify epigenetic instability. A low value for parameter ($\eta_{0}$) represents a high level of epigenetic instability.

### Model for optimizing intermittent treatment against the acquired drug resistance

Based on the paradigm of intermittent treatment [16], we proposed an optimal strategy for intermittent treatment against the acquired drug resistance. First, we denoted the therapeutic period as $\tau =\tau_{on}+\tau_{o\mathrm{ff}}$, where the parameters $\tau_{on}$ and $\tau_{o\mathrm{ff}}$ represent the therapeutic time with a high drug-dose (*c* = 0 . 9) and the drug holiday (*c* = 0), respectively. The drug-dose (*c*(*t*)) was rewritten as:


$$\begin{array}{ccc} c\left(t\right)=\{\begin{array}{ccc} 0.9, & & m\tau \leq t<(m+1)\tau -\tau_{o\mathrm{ff}}\\ 0.0, & & (m+1)\tau -\tau_{o\mathrm{ff}}\leq t<(m+1)\tau \end{array}. & & \end{array}$$ (25)

Here, the parameter *m* represents the m-th therapeutic period, and *N* is the set of natural numbers. The objective function of the optimization model for intermittent treatment was established as


$$\begin{array}{c} \begin{array}{c} \underset{\tau_{on},\tau_{o\mathrm{ff}}}{min}\;\underset{t\in (m\tau +\tau_{on},m\tau +\tau_{on}+\tau_{o\mathrm{ff}})}{max}\hat{Q}(t,c(t))\\ s.t.\;\hat{Q}(m\tau +\tau_{on},c(m\tau +\tau_{on}))<\hat{Q}(T_{0}). \end{array} \end{array}$$ (26)

The parameter $T_{0}$ is the preheating time (Supplementary Section Numerical Scheme in S1 Text), and $\hat{Q}$ is the total cell number. The preheating time is defined as the time point when the CTP distribution of all cells follows the steady-state distribution without perturbation from cytotoxic drugs. This concept was also used to simulate inflammation-related cancer development [5].

### Dynamics-based clustering method applied to scRNA-seq data of drug tolerance in lung cancer

**Preprocessing of scRNA-seq data.** First, we preprocessed the scRNA-seq data using the R package Seurat (v5.0.1) [17]. According to the quality criteria from the literature [11], cells were filtered using the range of number of genes per cell (nFeature_RNA). Then, ‘NormalizeData’ function was then utilized to normalize single-cell expression data with the default parameters [11]. The highly variable genes were determined using the ‘FindVariableFeatures’ function with the ‘vst’ method. We then performed transformation of these data using the ‘ScaleData’ function and principal component analysis of the scaled data using the ‘RunPCA’ function.

**Identifying cell clusters from the data across different drug treatment time points for PC9 cells.** Based on the scRNA-seq data across different drug treatment time points (see the section on Collection of data from the published literatures), we utilized the Seurat function “FindClusters” to identify clusters of cells. In these data, the dimension of the principal components (PCs) and cluster resolution were set to 12 and 1.0, respectively. We calculated the gene scores of the gene sets associated with cell proliferation and epigenetic instability. Here, the gene sets represent cells in the G2/M phase of the cell cycle. Because epigenetic instability is positively associated with stress response regulation [18], the gene sets associated with epigenetic instability are involved in two biological processes, namely, cellular responses to chemical stress (GO: 0062197) and oxidative stress (GO: 0034599). Leveraging the model-identified features of the three cell types, we clustered the drug-naïve cells (Clusters 1, 2, 5, 6, and 9), DTP cells (Clusters 0, 3, 7, and 10), and drug-resistant cells (Clusters 4 and 8) (Fig 4a and Fig Ba in S1 Text). Clusters 1, 2, 5, 6, and 9 are clustered as drug-naïve cells because these clusters showed low gene scores in the gene set of epigenetic instability and untreated cells (D0). Clusters 0, 3, 7, and 10 showed high gene scores in the gene set of epigenetic instability and low gene scores in the gene set of cell proliferation and were thus clustered as DTP cells. Clusters 4 and 8 were treated cells and showed high gene scores in the gene set of cell proliferation and low gene scores in the gene set of epigenetic instability; thus, these clusters were clustered as drug-resistant cells.

**Identifying cell clusters from the data across drug holidays for PC9 cells.** Based on the scRNA-seq data across drug holidays, we also used Seurat to identify cell clusters. Here the dimension and resolution were set to 15 and 1.2, respectively (Fig Bb in S1 Text). Using the Seurat function “FindMarkers” with the default parameters, we identified the top 20 differentially expressed genes among drug-naïve cells, DTP cells, and drug-resistant cells in the PC9 cell population treated with different drugs for different durations. The top 20 differentially expressed genes of each cell type were considered as the marker gene set for that cell type. We used the marker gene set of the three cell types to identify drug-naïve cells, DTP cells, and drug-resistant cells in the PC9 cell dataset for drug holidays. Clusters 2, 3, 4, 6, 7, and 10 exhibited high scores in the marker gene set for drug-naïve cells and were classified as drug-naïve cells. Clusters 8 and 10 exhibited high scores in the marker gene set for DTP cells and were classified as DTP cells. Clusters 0, 1, 5, 9, and 12 exhibited high scores in the marker gene set for drug-resistant cells and were thus classified as drug-resistant cells.

## Results

### Overview of the multiscale mathematical model of epigenetic population evolution

To uncover the potential mechanisms of acquired drug resistance, we developed a multiscale mathematical model of epigenetic population evolution in cancer. This model integrates epigenetic inheritance at the microscale level with cellular behaviors at the mesoscopic level. At the epigenetic level, we characterized epigenetic dynamics by modeling the cell proliferation process based on the previous study, which used Beta distribution [13] to represent the stochastic partitioning of epigenetic information from the mother cell. At the mesoscale level, similar to a previous work on describing cell behavior with a PDE model [7], we constructed a PDE model that integrates cell behaviors, such as proliferation and apoptosis, with epigenetic and phenotypic information. Finally, we developed a multiscale model to couple cell behavior with epigenetic inheritance. Then we derived a probability density function model of phenotype flux to represent phenotype changes dependent on the epigenetic level (see details in Materials and Methods).

The evolution of acquired drug resistance is quantified using the size of the cell population and the ratio of three types of cells: drug-naïve cells, DTP cells, and drug-resistant cells. Although a phenotype evolution model has previously been developed using integro-differential equations to quantify the three types of cells and capture the evolution of drug resistance [7], our model further incorporates more details of epigenetic dynamics during the proliferative phase of cells and utilizes measurable epigenetic information (CTP) to quantify cell proliferation ability. Moreover, compared with the probability density function for epigenetic dynamics directly developed using the Fokker–Planck equation [9], our model (Eqs (17) and (18)) that describes single-cell epigenetic dynamics is not only rational but also provides continuity and interpretability between population dynamics at the macroscale and epigenetic dynamics at the microscale.

The evolution of the cell population is simulated in a discrete time, following the algorithm illustrated in Fig 2 and Fig H in S1 Text. Over the time interval between two successive time instants *t* and *t* + *Δt*,we first allow each cell *i* either to proliferate, undergo apoptosis, or remain in a quiescent state according to the respective probabilities $P(z,\hat{Q},Q)\Delta t$, *A* ( *y* , *c* , *Q* ) *Δt*, and $\left(1-P(z,\hat{Q},Q)\Delta t-A(y,c,Q)\Delta t\right)$. If the cell undergoes cell division, it is replaced by two daughter cells, and the CTP state of each daughter cell is obtained through the inheritance probability function *p* ( *x* , *z* )  (Fig 2). The phenotype *y* of two daughter cells is inherited from the mother cell. If the cell is in the resting phase, the phenotype *y* for a single cell is updated using SDE (Fig 2, see details in the Section 2 in S1 Text).

### A data-driven multiscale model identifies distinctive features of the DTP cell subpopulation in PC9 cells

**Fig 3:**
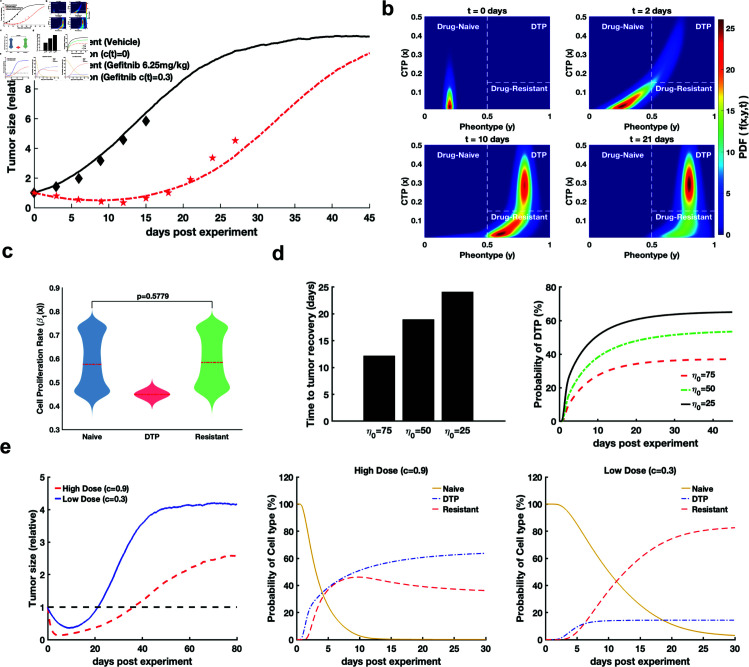
Reconstruction of epigenetic population dynamics and identification of dynamical features of DTP cell subpopulations. a. Time course of the relative tumor size. The black diamonds and red pentagrams show the data from the vehicle group and the group treated with gefitinib, respectively. The black solid line and red dashed line represent the numerical simulation results for the vehicle and treatment groups, respectively. b. Single-cell epigenetic dynamics on different days. The cell types were defined as drug-naïve cells  ( *y* < 0 . 5 ) , DTP cells  ( *y* > 0 . 5 , *x* > 0 . 15 ) , and drug-resistant cells  ( *y* > 0 . 5 , *x* < 0 . 15 )  (*x* denoted as *CTP*, and *y* denoted as the phenotype associated with drug-induced apoptosis). c. Violin plot of cell proliferation for the three cell types. This plot shows the proliferation rates of the three cell types determined by our models. The red dashed line is the median, and the significance was determined by a two-sided, *Wilcoxon* rank-sum test. d. Histogram of the time of tumor recovery and time course of the probability of cell types being affected by different epigenetic noise ($1∕\eta_{0}$) at high doses of cytotoxic drugs $\left(c(t)=0.9,t>T_{0}\right)$. e. Time course of the relative tumor size and the probability of cell types at high doses of cytotoxic drugs $\left(c(t)=0.9,t>T_{0}\right)$ and low doses of cytotoxic drugs $\left(c(t)=0.3,t>T_{0}\right)$. The yellow, blue and red lines represent the probabilities of cell types characterizing drug-naïve, DTP, and drug-resistant cells, respectively. All parameters are shown in Table A in S1 Text. Significance test was performed by a two-sided Wilcoxon rank-sum test.

To assess the ability of the model to recapitulate the experimental observations of PC9 cell lines treated with gefitinib, as depicted in Datasets 1 and 2, we simulated the model over a 60-day period during cytotoxic drug treatment ($c(t)=0.3,t>T_{0}$) (Fig 3a). Dataset 1 is from in vitro cell line experiments, measuring the distribution of CTP in PC9 cells both without gefitinib intervention and after seven days of gefitinib exposure. We used Dataset 1 to estimate the parameters $\phi_{0},\phi_{1},\alpha ,n$ and $\eta_{0}$ related to the epigenetic scale in our model. Dataset 2 consists of in vivo tumor cell data from mice. The control group measured the relative tumor volume without gefitinib intervention, while the experimental group measured the relative tumor volume at different time points after gefitinib injection. We used this data to estimate the parameters $\beta_{0},\gamma_{0},\beta_{10},a_{1},a_{2}$, and $a_{3}$ related to the cellular scale in our model. Additionally, we performed parameter sensitivity and identifiability analyses (Fig I, Fig J and Fig K in S1 Text; see details in Parameters estimation in S1 Text).

Based on numerical simulations, the relative size of the tumors decreased from 1 to approximately 0.3 after 10 days of treatment. Subsequently, the tumor size started to recover to its initial size before treatment on approximately day 20 and continued growing until it reached a stable size of approximately ten times its initial size on day 45. The population dynamics of PC9 cells, as simulated by our model, align well with the observed data for the tumor volume in mice transplanted with PC9 cells treated with gefitinib ($R_{vehicle}=0.94,R_{treatment}=0.89$). This agreement demonstrates that our model effectively predicts the dynamics of tumor cells after treatment with gefitinib at the population level.

Furthermore, we reconstructed the epigenetic dynamics at the single-cell level during treatment with a cytotoxic drug ($c(t)=0.3,t>T_{0}$). In the epigenetic-phenotypic space, we proposed a mathematical description of epigenetic inheritance for the three types of cells: drug-naïve cells (*y* < 0 . 5), DTP cells (*y* > 0 . 5 , *x* > 0 . 15), and drug-resistant cells (*y* > 0 . 5 , *x* < 0 . 15). Specifically, drug-naïve cells were defined based on their sensitivity to apoptosis induced by the cytotoxic drug, ensuring that their phenotype (*y*) was less than 0.5. Based on the previous statement that a high CTP is correlated with a low net growth rate [9], DTP cells are those with a CTP (*x*) greater than 0.15 (Fig Cc in S1 Text) and a phenotype (*y*) greater than 0.5. Drug-resistant cells are those with a CTP (*x*) less than 0.15 but a phenotype (*y*) greater than 0.5. With the persistent high-dose drug treatment, DTP cells emerged around day 2. By the day 10, the phenotype of drug-naive cells had transformed into a bimodal distribution of DTP cells and drug-resistant cells, and by the day 21, the population density of DTP cells was higher than that of drug-resistant cells (Fig 3b). Subsequently, the drug-naïve cell type disappears, the cell population predominantly exhibits a drug-resistant phenotype, and the tumor recovers to its initial size and then continues growing to a stationary size. According to the hypothesis stated in [9], the distribution of the cell proliferation rate $\beta_{1}(x)$ for the three cell types determined by our models (Fig 3c) showed that the proliferation rate of DTP cell subpopulations was lower than that of the other cell types, and that the difference between drug-naïve cells and drug-resistant cells was trivial. Before the cytotoxic drug treatment, drug-sensitive cells exist as two subpopulations with different proliferative capacities. During the initial phase of drug exposure, most of the high-proliferative subpopulation is eliminated, while the low-proliferative subpopulation transitions into DTP cells to survive. With prolonged exposure to the cytotoxic environment, some DTP cells evolve into drug-resistant cells with high proliferative capacity, leading to the formation of a high-proliferative subpopulation (Fig 3c). In addition, our model simplifies the probability functions of the CTP state for each cell and presents the survival curves of different fixed CTP states (Fig D in S1 Text), which are qualitatively in agreement with published work [9].

Moreover, to identify the factors regulating cell phenotype selection at the epigenetic level, we used both a multiscale model and a single-cell model for various levels of epigenetic noise ($1∕\eta_{0}$) at high doses of cytotoxic drugs ($c(t)=0.9,t>T_{0}$). During treatment with a high cytotoxic drug dose, we observed that a high level of epigenetic noise (low $\eta_{0}$) results in an increase in the time required to restore the initial tumor volume (Fig 3d). According to the results of the numerical simulations using the single-cell model, a high level of epigenetic noise corresponded to a high probability that the cell type is in the DTP state (Fig 3d). High epigenetic noise represents high epigenetic instability (see Materials and Methods). The results demonstrated that high epigenetic instability is a key factor in the dominance of DTP cell subpopulations.

To further explore whether adjusting the dose of the cytotoxic drug adaptively is effective against acquired resistance, we used our models to simulate a high dose of the cytotoxic drug ($c(t)=0.9,t>T_{0}$) and a low dose of the cytotoxic drug ($c(t)=0.3,t>T_{0}$). Through numerical simulations with the multiscale model, the results showed that the group treated with a low cytotoxic drug dose reached the initial size faster than the group treated with a high cytotoxic drug dose (Fig 3e). In constrast, the numerical results simulated at the microscale illustrate that cells were mostly characterized as being in the DTP state under high cytotoxic drug doses, whereas drug resistance dominates under low cytotoxic drug doses (Fig 3e). This result suggested that a low cytotoxic drug dose could induce large drug-resistant cell populations.

### Prediction of previously unrecognized biological features of drug tolerance using scRNA-seq data

**Fig 4:**
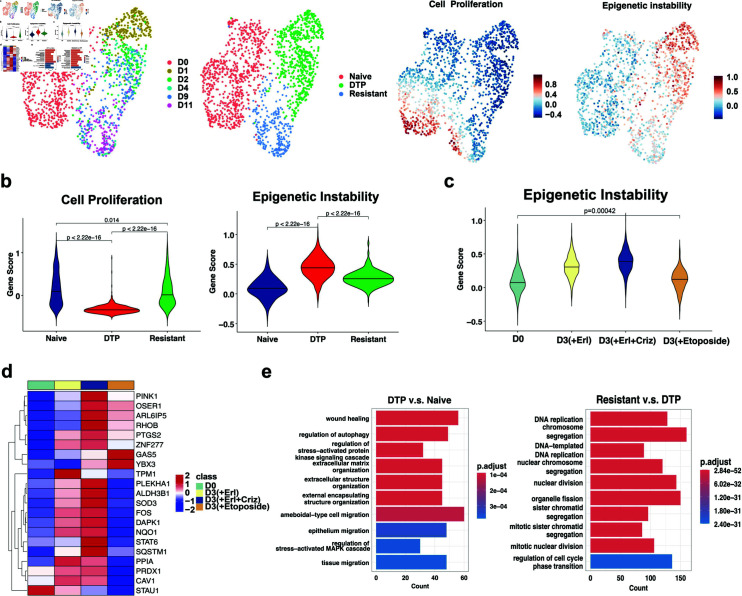
Prediction of new biomarkers of drug tolerance using scRNA-seq data. a. UMAP representation of PC9 cells at different treated days, with different cell types, and with different gene scores of biological processes. b. Violin plot of the gene score for the cell proliferation process and epigenetic instability. c. Violin plot of the gene scores of gene sets associated with epigenetic instability for the different drug treatment over three days of treatment. The black solid line is the median and the significance was determined by a two-sided Wilcoxon rank-sum test. d. Average gene expression of gene sets associated with epigenetic instability for the different drug treatments (Erl: erlotinib, Criz: crizotinob). The color bar represents the gene expression level. e. GO enrichment analysis of the differentially expressed genes between two different cell types. The color bar represents the adjusted p-value.

To validate the features of DTP cell subpopulations identified from our model, we analyzed a scRNA-seq dataset collected over five days (D1, D2, D4, D9 and D11) that characterizes the drug tolerance of lung cancer cells treated with erlotinib [11]. We performed uniform manifold approximation and projection (UMAP) dimensionality reduction of these individual cells using the Seurat R package, enabling the visualization of cells in a two-dimensional space based on their similar gene expression profiles (Fig 4a). By leveraging the model-predicted features of the DTP state and calculating the gene score for single cells using a gene set associated with cell proliferation and epigenetic instability (Fig 4a), we were able to cluster the cells into three groups, i.e., drug-naïve, DTP, and drug-resistant (see Materials and Methods), which were consistent with the original study that defined these cell states by biologists [11]. As expected, we observed significant differences in the cell proliferation ability and epigenetic instability between DTP and naïve or resistant cells, whereas the difference between drug-naïve cells and drug-resistant cells was less pronounced (Fig 4b). These results indicate that cell proliferation ability and epigenetic instability are indeed critical dynamic features of DTP cells.

Further analysis of different types of drug treatments on PC9 cell lines revealed a high level of epigenetic instability after treatment for more than three days, indicating that cancer cells treated with different drugs exhibit high epigenetic instability to tolerate drug cytotoxicity (Fig 4c). Given the importance of epigenetic instability reflected by both our model’s prediction and the scRNA-seq data, we further sought to identify the molecular features underlying the epigenetic instability, providing potential practical biomarkers for therapeutic targets. Interestingly, we identified upregulated genes associated with epigenetic instability after different drug treatments, particularly the genes TPM1, CAV1, and PPIA, which were highly enriched after three days of treatment with erlotinib (Fig 4d). In addition, high expression of GAS5 and YBX3 was also observed in the gene set associated with epigenetic instability after treatment with etoposide (Fig 4d). These upregulated genes may be potential biomarkers for overcoming drug tolerance. Moreover, the distinctive upregulation of genes associated with epigenetic instability after different drug treatments implies that drug tolerance is highly specific (Fig 4d), which is related to the specificity of CTP for drugs [8].

Additionally, to identify other underlying features of the DTP state, we performed a GO enrichment analysis of the differentially expressed genes between DTP and drug-naïve cells (Fig 4e). The result revealed two biological processes associated with DTP cells—the regulation of autophagy [19] and the MARK pathways associated with cell apoptosis [20]—which were not previously detected in a static data analysis [11]. Moreover, we predicted that cell migration is a potential feature of the DTP state given the enrichment of three biological processes: wound healing, ameboidal-type cell migration, and epithelial migration (Fig 4e). The GO enrichment analysis of differentially expressed genes between drug-resistant and DTP cells revealed that the predominant difference between these two cell types was the regulation of cell proliferation, such as DNA replication and mitotic nuclear division (Fig 4e).

### An optimal schedule of intermittent treatment for acquired drug resistance

**Fig 5:**
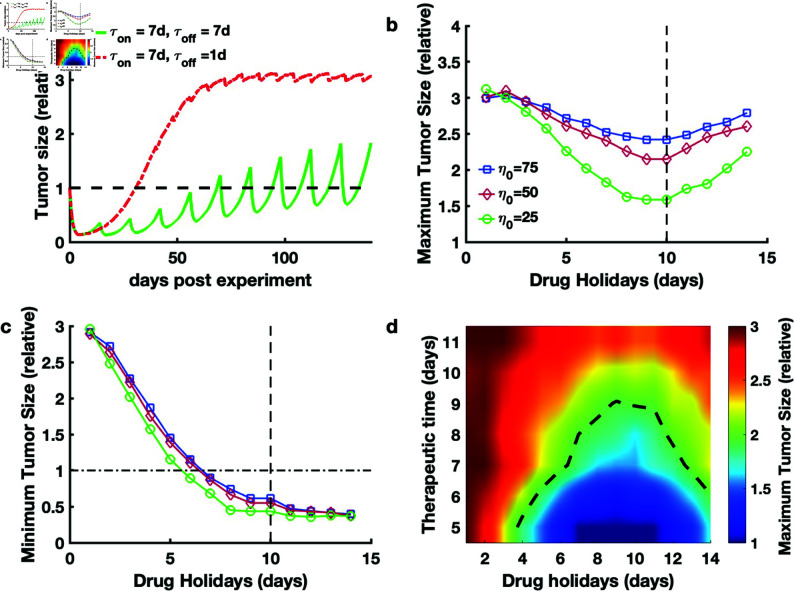
Population dynamics with different drug holidays upon exposure to a high dose of a cytotoxic drug. a. Time course of the relative size of tumor cells obtained with different drug holidays after seven days of treatment. The red and green lines represent drug holidays of seven days and one day, respectively. b. Maximum tumor size in the stable periodic dynamics with different drug holidays and epigenetic noise $\left(1∕\eta_{0}\right)$ when the drug treatment period was set to 7 days. c. Minimum tumor size under stable periodic dynamics with different drug holidays and epigenetic noise $\left(1∕\eta_{0}\right)$. d. Heatmap of the maximum tumor size under stable periodic dynamics when the epigenetic noise $\left(1∕\eta_{0}\right)$ was taken as the default value in Table A in S1 Text. The color bar represents the maximum tumor size, and the dashed line represents the threshold of the maximum tumor size at which the tumor size becomes twice compared to its initial size. Drug holidays and therapeutic time represents the drug-free period within a treatment cycle $\left(\tau_{o\mathrm{ff}}\right)$) and the drug administration period within a treatment cycle $\left(\tau_{on}\right)$, respectively. All parameters are presented in Table A in S1 Text.

In an experiment in which DTP cell subpopulations were induced by targeting antiapoptotic Bcl-2 proteins, a longer drug holiday of 6 days allowed recovery of the effectiveness of treatment, whereas a short drug holiday of 1 day resulted in less sensitivity to drug treatment [21]. This study suggested the possibility of intermittent therapy against acquired resistance; therefore, we investigated the nature of intermittent therapy against acquired resistance through a multiscale model. We performed our multiscale model to simulate the intermittent treatment case in which tumor cells were exposed to a high dose of a cytotoxic drug for 7 days ($\tau_{on}=7days$) and then not treated for 7 days ($\tau_{o\mathrm{ff}}=7days$). The numerical results showed that the tumor size was forced to exhibit stable periodic dynamics, indicating that the cancer cell population was sensitive to treatment (Fig 5a). When the drug holiday was decreased to 1 day ($\tau_{o\mathrm{ff}}=1day$), the tumor size reached a stationary level three times as larger than the initial tumor size, illustrating that the cancer cell population was tolerant to treatment. (Fig 5a). These numerical results indicate that our model recovers the experimental observations of long drug holidays [21].

Furthermore, we determined the optimal intermittent treatment based on Eq (26). Before stating the optimal schedule, we proposed an explanation for Eq (26), i.e., finding the minimum of the maximum tumor size to determine drug holidays ($\tau_{o\mathrm{ff}}$) when the therapeutic time ($\tau_{on}$) was set to satisfy the constraint of tumor size. When the therapeutic time ($\tau_{on}$) was set to be 7 days and the epigenetic noise ($1∕\eta_{0}$) was the default value, we performed our model with different drug holidays and analyzed the stable-period dynamics for the maximum and minimum tumor sizes during the therapeutic period. The maximum tumor size reached a minimum value when the drug holidays ($\tau_{o\mathrm{ff}}$) was set to 10 days (Fig 5b), whereas a minimum tumor size remained below the initial size (Fig 5c) only if the drug holidays was sufficiently long. When $\tau_{on}$ was set to 11 days, the minimum value of the maximum tumor size increased to approximately two times greater than that obtained when $\tau_{on}$ was set to 7 days (Figs S5a and S5b in S1 Text). Then, we identified the optimal area for the therapeutic time ($\tau_{on}$) and the drug holidays ($\tau_{o\mathrm{ff}}$) when the epigenetic noise ($1∕\eta_{0}$) was fixed to the default value (Fig 5d and Fig Ec in S1 Text). Moreover, the minimum value of the maximum tumor size also increased with decreases in epigenetic instability when both $\tau_{on}$ and $\tau_{o\mathrm{ff}}$ were fixed (Fig 5b), indicating that the epigenetic instability could be related to the effectiveness of intermittent treatment. These results provide a quantitative schedule for intermittent treatment to avoid treatment failure.

### Prediction of the epigenetic instability dynamics during intermittent treatment using a single-cell model and scRNA-seq data

**Fig 6:**
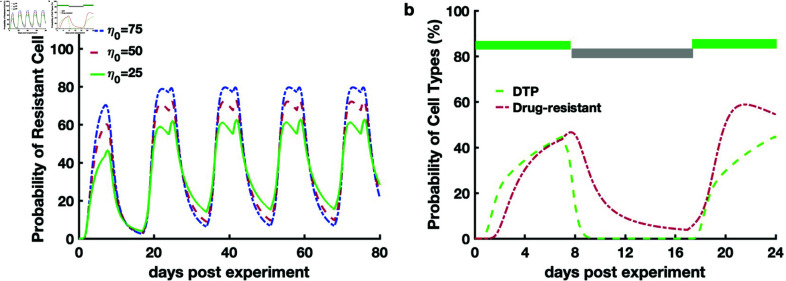
Single-cell phenotypic dynamics with drug holidays. a. Time course of the probability of resistant cells with different epigenetic noise ($1∕\eta_{0}$) with drug holidays set to 10 days. The different colored lines represent different levels of epigenetic noise ($1∕\eta_{0}$). b. Time course of the probability of DTP and resistant cells when the epigenetic noise ($1∕\eta_{0}$) remain unchanged at the second drug treatment ($\eta_{0}=25$). The green and gray bars represent the therapeutic time $\left(\tau_{on}=\text{7 days}\right)$, and drug-free time ($\tau_{o\mathrm{ff}}=\text{10 days})$, respectively. All parameters are provided in Table A in S1 Text.

The identified single-cell epigenetic dynamics showed that the ability of intermittent treatment to regulate cancer drug resistance is characterized by an oscillating probability of drug-resistant cells emerging (Fig 6a). As the epigenetic noise ($1∕\eta_{0}$) decreases, the probability of drug-resistant cells increases to 80% (Fig 6a). This result suggested that low epigenetic instability enables tumor cells to become drug-resistant cells. In addition, our results (Figs 5b and 5c) showed an increase in tumor size when epigenetic instability decreased. Therefore, decreased epigenetic instability might be a possible reason for treatment failure. Additionally, we decreased the inheritance probability of a high CTP level for daughter cells by decreasing the parameter *α*. The result (Fig L in S1 Text) showed a higher ratio of drug-resistant cells, suggesting that low level of CTP inheritance could be another factor leading to a higher resistant cell fraction. After a 10-day drug-free period, the proportion of drug-resistant cells observed at the second therapeutic time point of 7 days was still higher than that at the first therapeutic time point of 7 days, while the proportion of DTP cells remained unchanged. This suggests that the higher resistant cell fraction is not solely caused by reducing the noise, but may be caused by the persistence of a small number of resistant cells following the 10-day drug-free period (Fig 6b). Moreover, we found that when cell proliferation was inhibited, the probability of DTP cell subpopulations emerging remained unchanged (Fig Ee in S1 Text), although the maximum tumor size decreased (Fig Ed in S1 Text).

**Fig 7:**
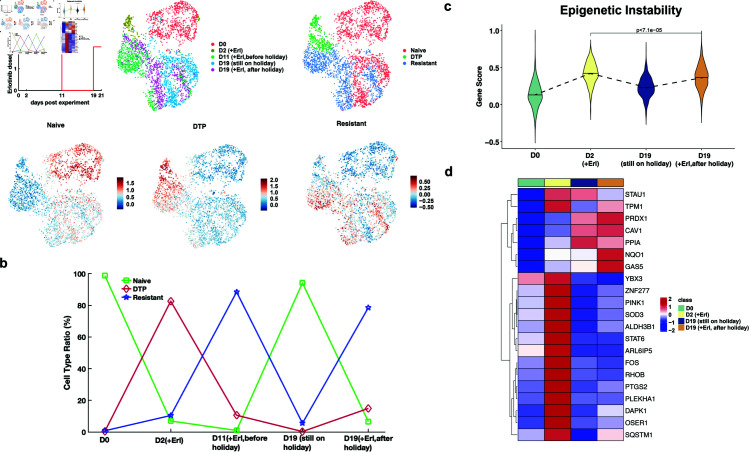
Differences associated with epigenetic instability before and after drug holidays. a. UMAP representation of PC9 cells on different treatment days with drug holidays and for different cell types. The color bar represents the gene score of gene sets associated with the top 20 differentially expressed genes for different cell types. b. Ratio of different cell types on the different treatment days. The green squares, red diamonds, and blue pentagrams represent the drug-naïve, DTP, and drug-resistant cells, respectively. c. Violin plot of the gene scores of gene sets associated with epigenetic instability on different treatment days. The black solid line is the median, and significance was determined by a two-sided Wilcoxon rank-sum test. d. Average expression levels of genes associated with epigenetic instability at different treatment times. The color bar represents the gene expression level.

Although we proposed an optimal schedule for intermittent treatment against acquired drug resistance and identified the epigenetic instability as a potential factor affecting the effectiveness of intermittent treatment, it is unclear whether changes in epigenetic instability occur before and after drug treatment. By leveraging the scRNA-seq data of drug tolerance before and after drug holidays, we identified the potential biomarker genes associated with epigenetic instability resulting in cancer drug tolerance. We visualized all cells at different treatment times in a two-dimensional space, and identified three cell types based on the top 20 differentially expressed genes of drug-naïve, DTP, and drug-resistant cells (Fig 7a and Fig Bb in S1 Text; see Materials and Methods). The drug holidays represent withdrawal from erlotinib for 6 days after 11 days of erlotinib treatment. Through the analysis of the ratio of different cell types at different treatment time points, we observed that the ratio of DTP two days after drug holidays was lower than the ratio at the beginning of treatment for two days (Fig 7b). Interestingly, the ratio of drug-resistant cells two days after drug holidays reached a high level, similar to that detected 11 days after drug treatment. Compared with the model prediction that the low epigenetic instability enables tumor cells to become drug resistant (Fig 6a), we inferred that the level of epigenetic instability in cancer cell populations is reduced after drug holidays.

Furthermore, we performed a statistical analysis of the gene scores associated with epigenetic instability and discovered that the level of epigenetic instability two days after drug holidays was significantly lower than the ratio at the beginning of treatment for two days (Fig 7c). This result revealed that tumor cells may adapt to drug cytotoxicity during intermittent treatment. Additionally, as revealed based on the average expression of gene sets associated with epigenetic instability, the upregulated genes were completely distinct after drug holidays compared to those before drug holidays (Fig 7d). Compared with the ratios of cell types at different drug treatment time points, three genes, namely, PRDX1, NQO1 and GAS5, could be potential biomarkers associated with erlotinib tolerance.

## Discussion

**Fig 8:**
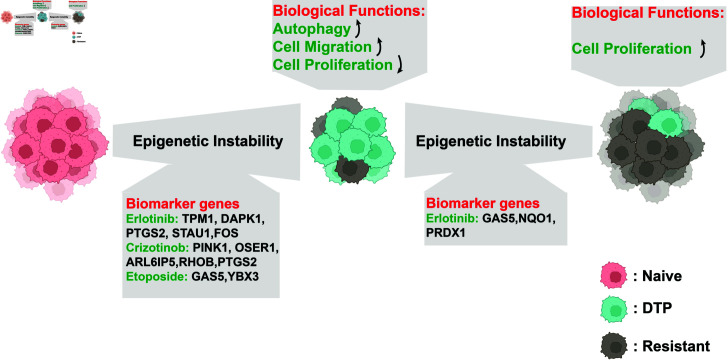
Summary of predictive biomarkers for the mechanism of acquired drug resistance identified in this study. This figure was created in BioRender. Wang, S. (2025) https://BioRender.com/f65x433.

The development of acquired drug resistance to therapy has been one of the current obstacles in the fight against cancer. Immediate evidence of the existence of acquired drug resistance is the experimental observation of DTP cell subpopulations upon exposure to a high dose of a cytotoxic drug [3]. However, tracking the fate of DTP cell subpopulations is challenging due to the heterogeneous gene markers of the DTP state [2]. Understanding the evolution of acquired drug resistance is a fundamental challenge. Although several types of experimental data are available from individual studies at separate biological scales [8,10,11], a systematic and dynamic understanding of the evolution of acquired drug resistance at multiple scales is lacking. In this study, we developed a multiscale computational framework based on published experimental data to explore the dynamic features of the evolution of acquired drug resistance and tested preliminary model predictions using scRNA-seq data. The main findings are summarized in Fig 8.

The important prediction of our model is that the dominance of DTP cell subpopulations is strictly related to high epigenetic instability (Fig 8). By leveraging the scRNA-seq data of drug tolerance in lung cancer, the DTP cells were clustered based on the model-predicted features. We further investigated the molecular features underlying the epigenetic instability of different drug-treated targets (Fig 8). Five genes, namely, TPM1, DAPK1, PTGS2, STAU1, and FOS, were identified as potential biomarkers of tolerance to erlotinib treatment. DAPK1 is known for its involvement in apoptosis and autophagy-associated cell death [22], and STAU1 is important for cell cycle progression and cell proliferation [23]. Potential biomarkers of tolerance to crizotinib treatment include the PINK1, OSER1, ARL6IP5, RHOB, and PTGS2 genes. High PINK1 expression promotes proliferation and chemoresistance in non-small cell lung cancer [24], and upregulated PTGS2 expression is associated with increased risk of colorectal cancer [25] and increased cell migration ability [26]. GAS5 and YBX3, as predicted biomarkers of tolerance to etoposide are related to cell proliferation [27,28]. Further GO enrichment analysis revealed two essential biological processes for DTP cell subpopulations: cell autophagy [19] and migration (Fig 8). Compared with DTP cells, a higher cell proliferation ability is an essential biological feature for drug-resistant cell subpopulations (Fig 8).

Our model-based analyses also suggested that low epigenetic instability enables tumor cells to become drug-resistant cells during the intermittent therapy (Fig 8). By leveraging the scRNA-seq data of drug holidays during erlotinib treatment, we validated the model prediction and identified three genes, namely, PRDX1, NQO1, and GAS5, as potential biomarkers associated with erlotinib tolerance (Fig 8). PRDX1 has been reported to be an inflammatory marker for colorectal cancer progression [29]. Upregulated NQO1 expression is associated with the risk of lung cancer [30] but promotes the efficacy of bioreductive anticancer drugs [31], suggesting the potential of multidrug intermittent therapy.

The tumor microenvironment also plays important roles in the acquired drug resistance [2]. By leveraging the correlation between epigenetic instability and five tumor immune subtypes [32], we explored the relationship between drug tolerance and the tumor immune microenvironment. By using the signature genes of five tumor immune subtypes [32], we calculated the Pearson correlation coefficients between epigenetic instability and the five tumor immune subtypes (Fig F in S1 Text), and found a positive correlation between the emergence of drug tolerance and the immune exclusion subtype, implying that immunotherapeutic tolerance may be related to the TIME with an immune exclusion subtype.

Our multiscale computational model is driven by the biological mechanisms of the DTP state and the collected data. Due to the limitations of the collected data in vitro cell line experiments, the findings from our model and scRNA-seq data analysis have not been validated in vivo in human dynamics. The parameters associated with the epigenetic level are derived from in vitro data, whereas the parameters at the cellular level are fitted using data from a mouse model. Since the cell lineage in Dataset 1 differs from the in vivo cells in the mouse, this discrepancy may affect parameter estimation at the population level. This limitation could be addressed if datasets were available from a single cell lineage at both the epigenetic and population levels. In addition, resistance to EGFR is mediated not only by non-genetic factors but also by genetic factors, such as extrachromosomally amplified DNA [33], which will be further investigated in the future work. Despite these potential limitations, our multiscale modeling framework and computational analytical approach using scRNA-seq data provide a novel avenue for predicting the dynamic features involved in the development of acquired drug resistance. Furthermore, the proposed model framework can be applied to study acquired drug resistance in other cancer types when relevant data are available.

## Supporting information

S1 Text Supplementary materials. (PDF)

## Acknowledgments

The numerical calculations in this paper have been done on the supercomputing system in the Supercomputing Center of Wuhan University. Fig 8 was created in BioRender. Wang, S. (2025) https://BioRender.com/f65x433.
