# Supplementary material for: Integrating multiscale mathematical modeling and multidimensional data reveals the effects of epigenetic instability on acquired drug resistance in cancer
Source: PLoS Comput Biol. 2025 Feb 14;21(2):e1012815. doi: 10.1371/journal.pcbi.1012815 (PMC11835379; doi:10.1371/journal.pcbi.1012815)
Supplement: S1 Text — (PDF) [file pcbi.1012815.s001.pdf]

Supplementary Materials

Integrating Multiscale Mathematical Modeling and Multidimensional Data Reveals the Effects of Epigenetic Instability on Acquired Drug Resistance in Cancer

Shun Wang, Jinzhi Lei, Xiufen Zou, Suoqin Jin

Content

SECTION 1 PARAMETERS ESTIMATION 2

SECTION 2 NUMERICAL SCHEME 7

SECTION 3 AGE-STRUCTURED MODEL OF EPIGENETIC DYNAMICS AT THE CELL PROLIFERATING PHASE 9

SECTION 4 SUPPLEMENTARY TABLES 11

SECTION 5 SUPPLEMENTARY FIGURES 12

Section 1 Parameters estimation

Parameters estimation using Dataset 1 and Dataset 2

All of the model parameters are obtained by fitting collected data from the published literatures. Because the CTP is drug-specific, all datasets consist of PC9 cell lines treated with gefitinib.

First, the parameters involved with the inheritance function such as $\phi_0, \phi_1, n, \eta_0, \alpha$ can be estimated by dataset 1. Assuming that the epigenetic state CTP distribution is homeostasis at the experimental data [1], we can estimate the parameters $\phi_0, \phi_1, n, \eta_0$ using the CTP distribution of naïve cells from dataset 1. The CTP distribution is written as $g(x,t)$ in Eq. (18), but the difficulty is the unknown parameters $\beta_1(x)$. Now, we further assume the $\beta_1(x)$ is independent on the CTP state $(\beta_1(x) = \beta_0)$, and the $g(x,t)$ in Eq. (18) can be expressed as

$$\frac{\partial g(x,t)}{\partial t} = 2\beta_0 \left(\int_0^1 g(z,t)p(x,z)dz - g(x,t) \right), \text{(S1)}$$

and the steady distribution of CTP state can be written as

$$g(x) = \lim_{t\to\infty} g(x,t) = \int_0^1 g(z)p(x,z)dz \,. \text{(S2)}$$

Thus, the steady distribution of CTP state can be obtained by Eq. (S2) and this distribution only depends on the inheritance function $p(x,z)$ controlled by the parameters $\phi_0, \phi_1, n, \alpha, \eta_0$. Because the parameter η_0 is related with the variance of beta distribution, we determined the parameter η_0 taken as 25 using the expectation and variance of the CTP distribution of naïve cells from dataset 1. Next, we simulate the steady distribution of CTP state and calculate the cumulative distribution using **Algorithm 1** in MATLAB to estimate the parameters of the function $\phi(z)$. The parameters in the function $\phi(z)$ such as $\phi_0, \phi_1, n, \alpha$ can be obtained as shown in Fig G. Further, the inheritance function also fit well to the CTP distribution of drug-released persister cells from the dataset 1 when we adjusted the parameter α to 6.0 (Fig G). These results show that the inheritance function is available to predicting the epigenetic dynamics of CTP.

Next, we determined the other parameters involved with cell behaviors and with gefitinib treatments. Using the vehicle group in the dataset 2, the parameters with cell proliferation and death can be estimated. The kinetic data of tumor volume is dealt with the time series of the tumor relative size compared to the tumor size at the initialized time (Fig. Ba). Referring that the PC9 cell lines are grown for 12 days [1], we set the preheating time T_0 as 12 days. For simplicity, when the β_{10} is set as zero, the parameters β_0 and γ_0 can be determined using the vehicle group in the dataset 2. Furthermore, we utilized the kinetic data of treatment with gefitinib in dataset 2 to estimate the

parameters v_0, c, γ_1 (Table A). The index R^2 quantifies the fit between model prediction and the experimental data as:

$$R^2 = 1 - \frac{\sum_{i=1}^{n}(u_i - \hat{u}_i)^2}{\sum_{i=1}^{n}(u_i - \bar{u})^2}, \text{(S3)}$$

where u_i is the value of the ith data point, $\hat{u}_i$ is predicted value of the ith data point and $\bar{u}$ is the mean of data point. R^2 reflects how well the model fits the sample data and Its value range is [0,1]. In addition, after the dose of the anticancer drug $c(t)$ is estimated using the dataset 2, the parameter k denoted as the relation between the dose of anticancer drug $c(t)$ and favoring CTP state α is determined (Figs. Ga-b). The results showing a low R^2 value for the treatment group in Fig. Ea indicate that cell proliferation is dependent on the CTP state $\left(R^2_{\text{vehicle}} = 0.91, R^2_{\text{treatment}} = 0.58\right)$.

Furthermore, we revised the above hypothesis that the higher CTP is, the slower cell proliferation is (Fig. Ec) [2], and reperformed the numerical simulation to determine the parameters such as $\beta_{10}, a_1, a_2, a_3$ (Table A). Based on the value of R^2 both in the treatment group with gefitinib and vehicle group, the determined parameters predicted well in both group (Fig. Ba and Fig. Cd), which implies that the revised hypothesis is rational.

Algorithm 1: Generating the steady distribution of CTP state (Eq. (S2))

Input: The parameters $\boldsymbol{\phi_0}, \boldsymbol{\phi_1}, \boldsymbol{n}, \boldsymbol{\eta_0}$

Output: Steady distribution vector of CTP state $(\boldsymbol{g_{1\times M}})$

1. Give an initial vector $\boldsymbol{g^0_{1\times M}} \sim \mathbf{U}(\mathbf{0}, \mathbf{1})$
2. **While** Convergence criterion is not fulfilled **DO**
3. Compute $\boldsymbol{\phi}(\boldsymbol{g^i_j}), \boldsymbol{\alpha}(\boldsymbol{g^i_j}), \textbf{and } \boldsymbol{\beta}(\boldsymbol{g^i_j})$ using Eq. (20) for each $\boldsymbol{g^i_j}$ $(\boldsymbol{j} = \mathbf{1}, \mathbf{2}, \dots, \boldsymbol{M}, \boldsymbol{i} = \mathbf{1}, \mathbf{2}, \dots)$
4. Update each $\boldsymbol{g^{i+1}_j} \sim \boldsymbol{Beta}\left(\boldsymbol{\alpha}(\boldsymbol{g^i_j}), \ \boldsymbol{\beta}(\boldsymbol{g^i_j})\right)$ $(\boldsymbol{j} = \mathbf{1}, \mathbf{2}, \dots, \boldsymbol{M})$ using random number obeying Beta distribution ("*betarnd*" function in Matlab)
5. **END WHILE**
6. **Return:** Steady distribution vector of CTP state $(\boldsymbol{g_{1\times M}})$

Note: $\mathbf{U}(\mathbf{0}, \mathbf{1})$ is a uniform distribution.

Parameter Identifiability

We utilized the Fisher information matrix (FIM) to analyze the identifiability to estimated parameters. The FIM is denoted as $F(\theta) = S^T(\theta)S(\theta)$, and $S(\theta)$ is the sensitive matrix as $\{s_{ij}\}_{M\times K}, s_{ij} = \frac{\partial \varphi}{\partial \theta_j}(t_i)$ where φ is the observable variable, M is the number of time points, and K is the number of analyzed parameters [3]. The parameters are local identifiable if the determinant of FIM is non-zero [4]. Using the eigenvalues and corresponding unite orthogonal eigenvectors of FIM, the identifiability of parameters is evaluated [5]. We ranked

all eigenvalues of FIM and found the identifiable parameter corresponding to maximum absolute value of element in the eigenvector.

With respect to Dataset 1, the parameters $\phi_0, \eta_0, \phi_1, \alpha,$ and n are estimated by this dataset. Using the CTP distribution of the drug-naïve cells, the parameters ϕ_0, η_0 are identifiable through calculating the data expectation as ϕ_0 and the data variance as η_0. The parameters ϕ_1, α, n are estimated by the CTP distribution of the drug-released cells. It is required to construct the sensitive matrix of these parameters using the chain rule as follows:

$$\frac{\partial p(x)}{\partial \phi_0} = \frac{\partial p(x)}{\partial a(z)}\frac{\partial a(z)}{\partial \phi_0} + \frac{\partial p(x)}{\partial b(z)}\frac{\partial b(z)}{\partial \phi_0},$$

where $p(x)$ is the steady distribution of CTP state exposure to cytotoxic drug. Because of $p(x) = \int_0^1 p(x,z)dz$, the $\frac{\partial p(x)}{\partial \phi_0}$ can be written as:

$$\frac{\partial p(x)}{\partial \phi_0} = \int_0^1 p(x,z;a(z),b(z))\big(\psi\big(a(z)\big) + \psi\big(b(z)\big) - 2\psi(a(z)+b(z))\big)\frac{(\alpha z)^n}{1+(\alpha z)^n}dz \quad (S4).$$

Herein, $\psi(x)$ is the digamma function denoted as $\psi(x) = \frac{d}{dx}\ln\Gamma(x)$.

Similarity, we also write the partial derivatives $\frac{\partial p(x)}{\partial \alpha}, \frac{\partial p(x)}{\partial n}$ as

$$\frac{\partial p(x)}{\partial \alpha} = \int_0^1 p\big(x,z;a(z),b(z)\big)\Big(\psi\big(a(z)\big) + \psi\big(b(z)\big) - 2\psi\big(a(z)+b(z)\big)\Big)\frac{nz(\alpha z)^{n-1}}{(1+(\alpha z)^n)^2}dz \quad (S5)$$

$$\frac{\partial p(x)}{\partial n} = \int_0^1 p\big(x,z;a(z),b(z)\big)\Big(\psi\big(a(z)\big) + \psi\big(b(z)\big) - 2\psi\big(a(z)+b(z)\big)\Big)\frac{(\alpha z)^n\ln(\alpha z)}{(1+(\alpha z)^n)^2}dz \quad (S6).$$

The sensitive matrix of parameter $\phi_1, \alpha,$ and n is denoted as $S(\phi_1, \alpha, n)$ as follows:

$$S(\phi_1,\alpha,n) = \begin{bmatrix} \frac{\partial p(x_1)}{\partial \phi_0} & \frac{\partial p(x_1)}{\partial \alpha} & \frac{\partial p(x_1)}{\partial n} \\ \frac{\partial p(x_2)}{\partial \phi_0} & \frac{\partial p(x_2)}{\partial \alpha} & \frac{\partial p(x_2)}{\partial n} \\ \vdots & \vdots & \vdots \\ \frac{\partial p(x_N)}{\partial \phi_0} & \frac{\partial p(x_N)}{\partial \alpha} & \frac{\partial p(x_N)}{\partial n} \end{bmatrix} \quad (S7)$$

where $x_1, x_2, \dots, x_N$ are data points from the CTP distribution of the drug-released cells. Through computing the eigenvalues of FIM $F(\phi_1, \alpha, n)$, we found that the three parameters are local identifiable because the eigenvalues of FIM $F(\phi_1, \alpha, n)$ is nonzero (Fig. Ia). Furthermore, using the eigenvectors of FIM $F(\phi_1, \alpha, n)$, the result illustrated that the parameter ϕ_1 has the strongest identifiability, followed by parameter α, while parameter n has relatively weaker identifiability (Fig. Ib). This result suggests that the Hill coefficient *n* is better to be determined based on prior knowledge whenever possible.

With respect to Dataset 2, the parameters $\beta_0, \gamma_0, \beta_{10}, a_1, a_2$ and a_3 are estimated by this dataset. For simplicity, when the dose of cytotoxic drug is set as zero, the differential equation on the total population size of cancer cell $\hat{Q}$ based on Eq. (14) is rewritten as

$$\frac{d\hat{Q}}{dt} = \beta_0\hat{Q}\left(1-\frac{\hat{Q}}{K}\right) - \gamma_0\hat{Q} + \beta_{10}\hat{Q}\left(1-\frac{\hat{Q}}{K}\right)\int_0^1 \frac{a_1x+(a_2x)^6}{1+(a_3x)^6}p_{ss}(x)dx,$$

where $p_{ss}(x)$ is steady distribution of CTP state without perturbation of cytotoxic drug. We denote the partial derivatives as $\xi_1 = \frac{\partial\hat{Q}}{\partial\beta_0}, \xi_2 = \frac{\partial\hat{Q}}{\partial\gamma_0}, \xi_3 = \frac{\partial\hat{Q}}{\partial\beta_{10}}, \xi_4 = \frac{\partial\hat{Q}}{\partial a_1}, \xi_5 = \frac{\partial\hat{Q}}{\partial a_2}, \xi_6 = \frac{\partial\hat{Q}}{\partial a_3}$ and write these differentiable equations as follows:

$$\frac{d\xi_1}{dt} = \hat{Q}\left(1-\frac{\hat{Q}}{K}\right) + \beta_0\left(1-2\frac{\hat{Q}}{K}\right) - \gamma_0 + \beta_{10}\left(1-2\frac{\hat{Q}}{K}\right)\int_0^1 \frac{a_1x+(a_2x)^6}{1+(a_3x)^6}p_{ss}(x)dx, (S8)$$

$$\frac{d\xi_2}{dt} = -\hat{Q} + \beta_0\left(1-2\frac{\hat{Q}}{K}\right) - \gamma_0 + \beta_{10}\left(1-2\frac{\hat{Q}}{K}\right)\int_0^1 \frac{a_1x+(a_2x)^6}{1+(a_3x)^6}p_{ss}(x)dx, \quad (S9)$$

$$\frac{d\xi_3}{dt} = \hat{Q}\left(1-\frac{\hat{Q}}{K}\right) + \beta_0\left(1-2\frac{\hat{Q}}{K}\right) - \gamma_0 + \beta_{10}\left(1-2\frac{\hat{Q}}{K}\right)\int_0^1 \frac{a_1x+(a_2x)^6}{1+(a_3x)^6}p_{ss}(x)dx, (S10)$$

$$\frac{d\xi_4}{dt} = \beta_{10}\hat{Q}\left(1-\frac{\hat{Q}}{K}\right)\int_0^1 \frac{x}{1+(a_3x)^6}p_{ss}(x)dx + \beta_0\left(1-2\frac{\hat{Q}}{K}\right) - \gamma_0$$

$$+\beta_{10}\left(1-2\frac{\hat{Q}}{K}\right)\int_0^1 \frac{a_1x+(a_2x)^6}{1+(a_3x)^6}p_{ss}(x)dx \ (S11),$$

$$\frac{d\xi_5}{dt} = \beta_{10}\hat{Q}\left(1-\frac{\hat{Q}}{K}\right)\int_0^1 \frac{6a_2^5x^6}{1+(a_3x)^6}dx + \beta_0\left(1-2\frac{\hat{Q}}{K}\right) - \gamma_0$$

$$+\beta_{10}\left(1-2\frac{\hat{Q}}{K}\right)\int_0^1 \frac{a_1x+(a_2x)^6}{1+(a_3x)^6}p_{ss}(x)dx \ S(12),$$

$$\frac{d\xi_6}{dt} = \beta_{10}\hat{Q}\left(1-\frac{\hat{Q}}{K}\right)\int_0^1 \frac{(a_1x+(a_2x)^6)(-6a_3^5x^6)}{(1+(a_3x)^6)^2}p_{ss}(x)dx + \beta_0\left(1-2\frac{\hat{Q}}{K}\right) - \gamma_0$$

$$+\beta_{10}\left(1-2\frac{\hat{Q}}{K}\right)\int_0^1 \frac{a_1x+(a_2x)^6}{1+(a_3x)^6}p_{ss}(x)dx \ (S13).$$

The initial condition of above differentiable equations $\xi_i(0) = 0 \ (i = 1,2,\ldots,6)$ because the initial condition is independent on these parameters. The sensitive matrix of these parameters is denoted as $S(\beta_0,\gamma_0,\beta_{10},a_1,a_2,a_3)$ as follows:

$$S(\beta_0,\gamma_0,\beta_{10},a_1,a_2,a_3) = \begin{bmatrix} \xi_1(t_1) & \xi_2(t_1) & \cdots & \xi_6(t_1) \\ \xi_1(t_2) & \xi_2(t_2) & \cdots & \xi_6(t_2) \\ \vdots & \vdots & \cdots & \vdots \\ \xi_1(t_N) & \xi_2(t_N) & \cdots & \xi_6(t_N) \end{bmatrix} (S14).$$

where $t_1, t_2, \ldots, t_N$ are data points from the total number of cancer cells at the vehicle group. By computing the eigenvalues of FIM $F(\beta_0, \gamma_0, \beta_{10}, a_1, a_2, a_3)$, we found that the six parameters are local identifiable because the eigenvalues of FIM $F(\beta_0, \gamma_0, \beta_{10}, a_1, a_2, a_3)$ is nonzero (Fig. Ja). Furthermore, though the eigenvectors of FIM $F(\phi_1, \alpha, n)$, the result showed that the combination of parameter γ_0 and β_0 has the strongest identifiability, followed by parameter β_{10}, while parameters $a_1, a_2,$ and a_3 has the weakest (Fig. Jb). By fitting the population dynamics data, the cell behavior associated parameters $(\beta_0, \beta_{10}, \gamma_0)$ exhibit higher identifiability compared to those epigenetic regulation(a_1, a_2, a_3). This observation underscores the importance of estimating epigenetic-level parameters using epigenetic data whenever possible. As demonstrated in Fig. 3, the results indicate that the model successfully recovers the dynamics of the real data, suggesting that the algorithm effectively finds a local minimum in the parameter space.

Parameter Sensitivity

We also utilized the Fisher information matrix (FIM) to analyze the sensitivity to estimated parameters and added the one figure on the sensitivity of estimated parameters. The classical sensitivity coefficient for an observable φ and parameter θ is $S = \frac{\partial \varphi}{\partial \theta}$, and the FIM is a measure of how the observable changes in response to infinitesimal changes in parameters [4]. Using the eigenvalues and corresponding unite orthogonal eigenvectors of FIM, if we assume that the eigenvalues denoted as λ_i are ordered so that $\lambda_1 > \lambda_2 > \cdots > \lambda_K$, then it follows that around the maximum the likelihood or the minimum the cost function that is used to determine the estimated parameters is most sensitive when the eigenvector denoted as $U_1^T \theta$ is varied and least sensitive when $U_K^T \theta$ is varied, and λ_i is a measure of this. We can regard $\mathcal{S}_{ij} = \lambda_i^{1/2} U_{ij}$ as the contribution of the parameter θ_j to varying $U_j^T \theta$ and thus $\mathcal{S}_j = \sum_{i=1}^{K} \mathcal{S}_{ij}^2$ can be regarded as a measure of the sensitivity of the system to θ_j. It is sometimes appropriate to normalize this and instead consider $\mathcal{F}_j = \frac{\mathcal{S}_j}{\sum_{i=1}^{L} \mathcal{S}_i}$.

At the epigenetic level, the observable variable is the steady distribution of CTP as $p(x)$ and the parameters ϕ_1, α, n are estimated by the CTP distribution of the drug-released cells. Through the eigenvalues and eigenvector of FIM $F(\phi_1, \alpha, n)$ to calculate the $\mathcal{F}_j$ for parameters ϕ_1, α, n, we found that the parameter ϕ_1 is most sensitive, followed by parameter α, while parameter n has the weakest sensitivity (Fig. Ka). At the population level, the observable variable is the total population size of cancer cells as $\hat{Q}$ and the estimated parameters are $\beta_0, \gamma_0, \beta_{10}, a_1, a_2$ and a_3. Sensitivity analysis illustrated that the parameters γ_0 and β_0 are most sensitive, followed by

parameter β_{10}, while parameters $a_1, a_2,$ and a_3 has the weakest sensitivity (Fig. Kb). Based on sensitive analysis, parameters $(\beta_0, \beta_{10}, \gamma_0)$ associated with cell behavior demonstrate higher sensitivity compared to those (a_1, a_2, a_3) related to epigenetic regulation.

Section 2 Numerical scheme

Multiscale Model. The proposed multiscale model is established using agent-based stochastic simulation. The schematic framework is summarized in Fig. H.

The scheme initializes with a tissue system of $\hat{Q}_0$ cells and the CTP state distribution of these cells is set as obeying uniform distribution at the interval (0,1) $\left(x \sim U(0,1)\right)$. The distribution of phenotype y for initial cells is set as obeying the normal distribution with expectation value taken as $y_0(0)$ and variance value taken as 0.01 $\left(y \sim N(y_0(0), 0.01)\right)$. In addition, we set the preheating time T_0 to perform the system under the condition without exposure to the cytotoxic drug.

In numerical simulations, the system is initialized with initial cell numbers, CTP state distribution, and phenotype, and preheated under conditions without cytotoxic drugs. We run the program with $c(t) = 0,\ 0 < t < T_0$ using a time step $dt = 0.04\ \text{days}$, so that the system reaches a stationary CTP state under no-drug condition. Then, we set the level of cytotoxic drug to a given dose starting from $t \geq T_0$. Data analysis is based on simulation data after $t \geq T_0$. Note the Eq. (2) for each cell, the dynamics of phenotype y is rewritten using stochastic differential equation as

$$dy = v\left(x, y, c(t)\right)dt + \sqrt{2\sigma}W(t), (S15)$$

where $W(t)$ is a wiener process.

The numerical scheme is shown below (Fig. H):

1. **System Initialization:** Set the time $t = 0$, preheating time T_0 and the step size $dt = 0.04\ \text{days}$. Initialize the system states, including the initial cell number $\hat{Q}_0$, the CTP state distribution of initial cells is set as obeying uniform distribution at the interval (0,1) $\left(x \sim U(0,1)\right)$, and the distribution of phenotype y for initial cells is set as obeying the normal distribution with expectation value taken as $y_0(0)$ and variance value taken as 0.01 $\left(y \sim N(y_0(0), 0.01)\right)$.
2. **Update cell epigenetic and phenotypic state:** For each cell in system

a) Calculate the proliferation rate β and apoptosis rate γ.

b) Determine the cell fate during the time interval $(t, t + \Delta t)$: The cell is removed (through apoptosis) with a probability $\gamma \Delta t$, or divides into two daughter cells with a probability $\beta \Delta t$, or stay at the resting phase with a probability $(1 - \beta \Delta t - \gamma \Delta t)$.
c) If the cell undergoes cell division, it is replaced by two daughter cells, and the CTP state of each daughter cell is obtained through the inheritance probability function $p(x, z)$. The phenotype y of two daughter cells is inherited from the mother cell.
d) If the cell is in the resting phase, the phenotype y is updated using Eq. (S15).

3. **Update the system:** Update the system with cell number, CTP states and phenotype of all surviving cells $\hat{Q}(t)$, and the dose of cytotoxic drug $c(t)$.
4. **Update the time:** Let $t = t + dt$, and either go to step 2 or terminate the simulation process.

Single-Cell epigenetic dynamics. The proposed epigenetic model for each cell using Eqs. (17)-(18) is numerically simulated using finite difference method.

We discretized the epigenetic-phenotypic space $(x, y) \in (0,1) \times (0,1)$ to $N \times M$ grid points, and denote discretized CTP step size as $\Delta x = x_{i+1} - x_i$ $(i = 1, 2, \ldots, N-1)$, discretized phenotypic step size as $\Delta y = y_{j+1} - y_j$ $(j = 1, 2, \ldots, M-1)$, and time step size as $\Delta t = t_{n+1} - t_n$. The inheritance function $p(x, z)$ can be discretized to the probability transition matrix $P = \{p_{ij}\}_{N \times N}$, and the integral operator is substituted using the inner product. So, the Eq. (18) can be simulated using finite difference method as:

$$\frac{g(x_i, t_{n+1}) - g(x_i, t_n)}{\Delta t} = 2\langle \beta_1(\boldsymbol{x}), P g(\boldsymbol{x}, t_n) \rangle - g(x_i, t_n) \langle \beta_1(\boldsymbol{x}), g(\boldsymbol{x}, t_n) \rangle$$
$$- g(x_i, t_n) \beta_1(x_i), \quad i = 1,2, \ldots, N.$$

Where $\langle \cdot \rangle$ represents the inner product, and $\boldsymbol{x} = [x_1, x_2, \ldots, x_N]^T$.

The Eq. (17) describes the conditional probability distribution of phenotype y with the fixed CTP state x. For each CTP state x_i $(i = 1,2, \ldots, N)$, $h(y, t_i; x_i)$ is denoted as the conditional probability distribution of phenotype y with the CTP state x_i. The Eq. (17) is rewritten using the finite difference method as

$$\frac{h(y_j, t_{n+1}; x_i) - h(y_j, t_n; x_i)}{\Delta t}$$
$$= \frac{v\left(x_i, y_{j-1}, c(t_n)\right) h(y_{j-1}, t_n; x_i) - v\left(x_i, y_{j+1}, c(t_n)\right) h(y_{j+1}, t_n; x_i)}{2\Delta y}$$
$$+ \sigma \frac{h(y_{j-1}, t_n; x_i) + h(y_{j+1}, t_n; x_i) - 2h(y_j, t_n; x_i)}{\Delta y^2} - h(y_j, t_n; x_i) \gamma\left(y_j, c(t_n)\right)$$
$$+ h(y_j, t_n; x_i) \langle \gamma(\boldsymbol{y}, c(t_n)), H(\boldsymbol{y}, t_n) g(\boldsymbol{x}, t_n) \rangle, j = 2,3, \ldots, M-1$$

The boundary condition is simplified as

$$h(y_M, t_n; x_i) = h(y_1, t_n; x_i) = 0, \forall n \in N^+, \forall i \in \{1,2,\dots,N\}$$

Where $H(y, t_n) = [h(\boldsymbol{y}, t_n; x_1), h(\boldsymbol{y}, t_n; x_2), \dots, h(\boldsymbol{y}, t_n; x_N)]^T$ and $h(\boldsymbol{y}, t_n; x_i) = [h(y_1, t_n; x_i), h(y_2, t_n; x_i), \dots, h(y_M, t_n; x_i)]^T$. The union distribution of epigenetic-phenotypic information $f(\boldsymbol{x}, \boldsymbol{y}, t_n)$ for a single cell at the time t_n is rewritten as

$$f(\boldsymbol{x}, \boldsymbol{y}, t_n) = H(\boldsymbol{y}, t_n) g(\boldsymbol{x}, t_n).$$

In the simulation, we set Δx as 0.01, Δy as 0.001, and Δt as $0.4\Delta y^2/\sigma$.

Section 3 Model derivation

Age-structured model of epigenetic dynamics at the cell proliferating phase

The mathematical model of epigenetic dynamics is developed based on a series of relevant published work [6–8]. At the cell proliferating phase, $s(t, a, x, y)$ is an age-structed quantify that represents the population of proliferating cells, and $a = 0$ is their time of entry into the proliferative state. The resting-phase cells can reenter the proliferative phase at a rate $\beta(x, Q, \hat{Q})$. Generally, the proliferative cells are assumed to undergo mitosis at a fixed time τ after entry into the proliferating compartment and to be lost at a rate μ during the proliferating phase [6,8]. Each normal cell generates two resting-phase cells at the end of mitosis. Thus, the age-structured model as shown in Fig. 2 follows as:

$$\frac{\partial s(t, a, x, y)}{\partial t} + \frac{\partial s(t, a, x, y)}{\partial a} = -\mu s(t, a, x, y) \ (t > 0, 0 < a < \tau) \ \text{(S16)}$$

$$P(x, \hat{Q}, Q) = 2 \int s(t, \tau, z, y) p(x, z) dz - \beta(x, Q, \hat{Q}) Q(t, x, y) \ (t > 0) \quad \text{(S17)}$$

The boundary condition at $a = 0$ is as follows:

$$s(t, 0, x, y) = \beta(x, Q, \hat{Q}) Q(t, x, y).$$

By integrating Eqs. (S16) and (S17) with the characteristic line method, we obtain the following equation (here we only show the result for long-term behavior):

$$s(t, \tau, x, y) = \beta(x, Q, \hat{Q}(t - \tau)) Q(t - \tau, x, y) e^{-\mu\tau} \ \text{(S18)}$$

Substituting $s(t, \tau, x, y)$ into the Eq. (6), we obtain

$$P(x, \hat{Q}, Q) = 2 \int \beta\left(z, Q, \hat{Q}(t - \tau)\right) Q(t - \tau, z, y) e^{-\mu\tau} p(x, z) dz$$

$$-\beta(x, Q, \hat{Q}) Q(t, x, y) \ \text{(S19)}$$

For simplicity, we assumed that the fixed time τ is set to zero so that Eq. (S19) is rewritten as the Eq. (7).

Stochastic differential equation of phenotype dynamics for a single cell at the quiescent phase

When a single cell is in the quiescent state, only changes in the phenotype (y) are considered. We rewrite the conditional probability equation of phenotype variable y (Eq. (17)) for a single cell at the quiescent phase as follows:

$$\frac{\partial h(y,t;x)}{\partial t} = -\frac{\partial}{\partial y}\left\{v\big(x,y,c(t)\big)h(y,t;x)\right\} + \sigma\frac{\partial^2}{\partial y^2}h(y,t;x), \text{(S20)}$$

where $\int_0^1 h(y,t;x)dy = 1$. The stochastic differential equation [9] of phenotype dynamics for a single cell at the quiescent phase follows as:

$$dy = v\big(x,y,c(t)\big)dt + \sqrt{2\sigma}W(t),$$

where $W(t)$ is a wiener process.

Section 4 Supplementary Tables

Table A Parameters in the multiscale model

Parameters	Biological description	Value	Unit	Reference Range	Source
β_0	Basic proliferation rate of tumor cells	0.4	day^{-1}	[0, 0.45]	[10]
β_{10}	Maximum proliferation rate of tumor cells	1.0	day^{-1}	[0,1.92]	[11]
a_1	Regulatory constant of CTP to cell proliferation	8.0	–	/	[12]
a_2	Regulatory constant of CTP to cell proliferation	9.0	–	/	[12]
a_3	Regulatory constant of CTP to cell proliferation	15.0	–	/	[12]
K	The carrying capacity of tumor cells	3×10^5	cells		[13]
γ_0	Natural apoptosis rate of tumor cells	0.4	day^{-1}	[0, 0.45]	[10]
γ_1	Natural apoptosis rate of cytotoxic drug	1.0	day^{-1}	/	[14]
σ	Size of the fluctuations in phenotype y	10^{-3}	–	/	[13]
v_0	cell stress-induced adaptation	2.5	–	[0.005, 3.0]	[13,14]
ϕ_0	Regulatory constant of inheritance function	0.063	–	/	[12]
ϕ_1	Regulatory constant of inheritance function	0.25	–	/	[12]
η_0	Noise fluctuation of altered CTP state	25	–	/	[12]
n	Regulatory constant of inheritance function	6.0	–	/	[12]
α_0	Basic favoring CTP without cytotoxic drug	4.0	–	/	[12]
k	Positive coefficient of favoring high CTP to drug-dose	6.67	–	/	/

Note: If the β_{10} is set as zero, the parameter β_0 is taken as 0.56 estimated by the vehicle group in the dataset 2. $\hat{Q}_0$ is set to be 1500 cells.

Section 5 Supplementary Figures

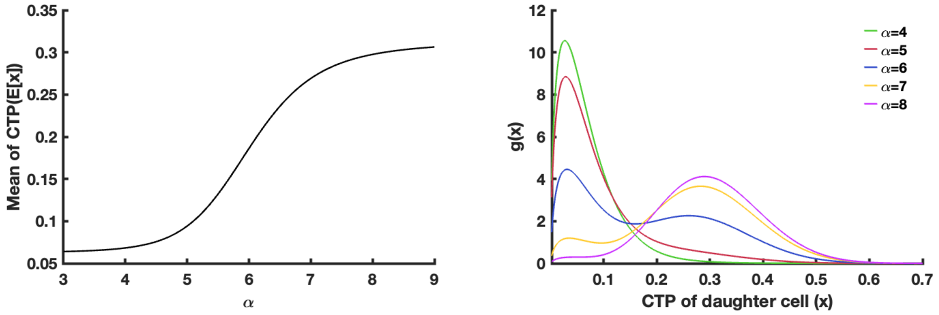

Fig. A. Degree of selective pressure favoring high CTP state (α) related with high dose-drug.

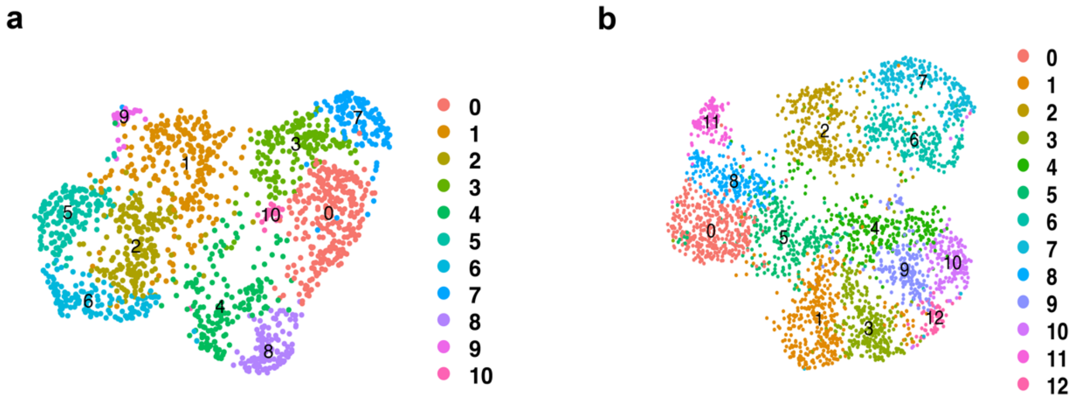

Fig. B. UMAP representation of cells by clusters. a. PC9 cells at the different treated days. **b.** PC9 cells at the treated days with drug holidays.

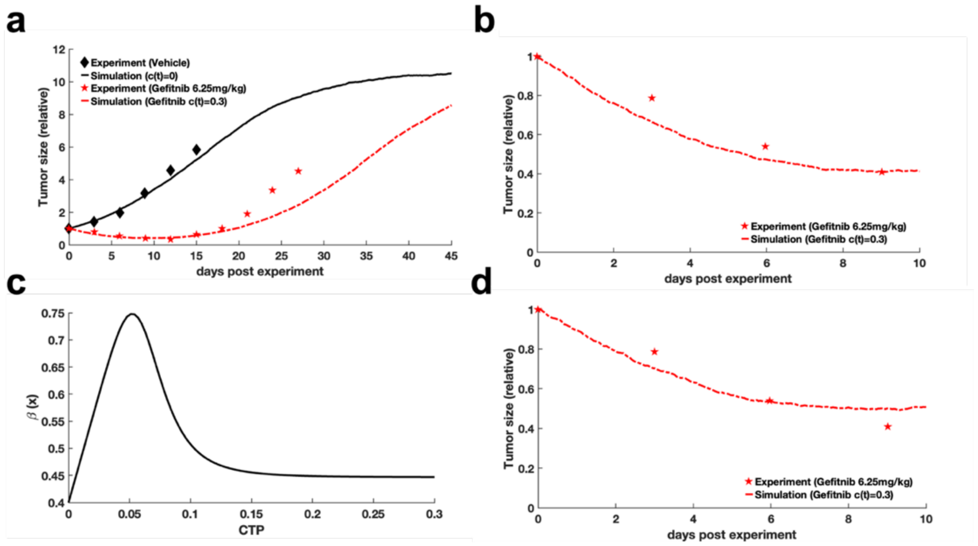

Fig. C. Comparison of population dynamics between simulation and real data from Dataset 2. a. Time course of tumor relative size when the β is independent on CTP state.

b. Time course of tumor relative size during the decay of the population $(R^2 = 0.91)$. **c.** Distribution of proliferating rate on the CTP state. **d.** Time course of tumor relative size during the decay of the population when the β is dependent on CTP state $(R^2 = 0.93)$**.** The black diamonds and red pentagrams are from vehicle group and group treated with gefitinib, respectively. The black solid line and red dash line represents the numerical simulation to vehicle and treatment group, respectively.

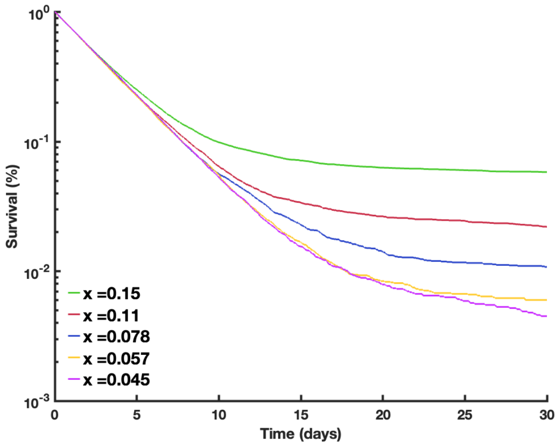

Fig. D. The decay of the population of various fixed-x clones. All fixed CTP state is referred to [14]. $v_0 = 1.0$, $c(t) = 0.3, t > T_0$. The parameters β_0, β_1 and γ_0 are taken as zero because cell proliferation and normal apoptosis are ignored in the published work [14]. The other parameters are default values in Table A.

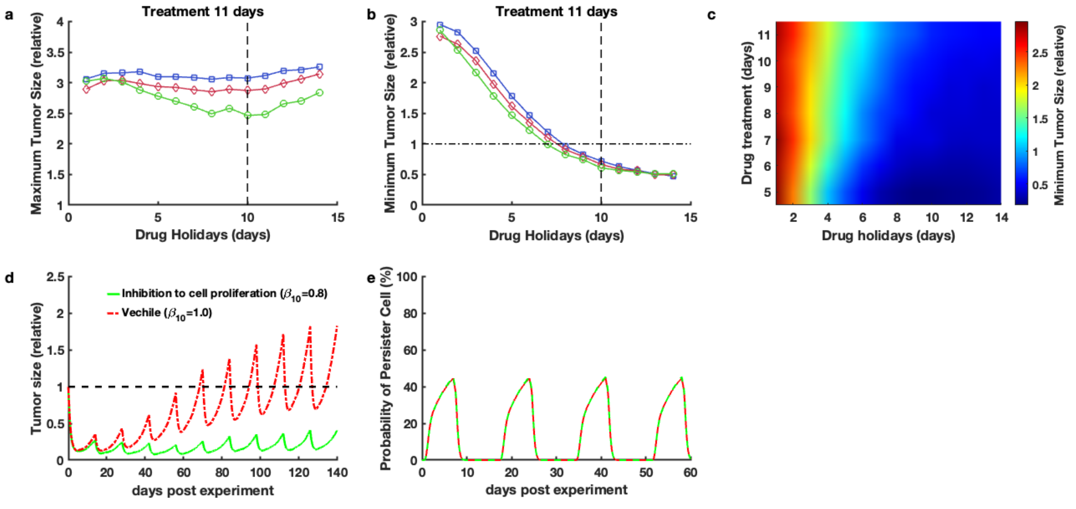

Fig. E. The population dynamics and cell phenotypic dynamics at different drug holidays upon exposure to high dose of cytotoxic drug. a. Maximum tumor size in the stable periodic dynamics with different drug holidays and epigenetic noise $(1/\eta_0)$ when drug treatment period set as 11 days. **c.** Minimum tumor size in the stable periodic dynamics with different drug holidays and epigenetic noise $(1/\eta_0)$ when drug treatment period set as 11 days. **c.** Heatmap of minimum tumor size in the stable periodic dynamics when the epigenetic noise (η_0) is taken as default value in Table A. The color bar

represents minimum tumor size. **d.** Time course of the tumor size with different cell proliferation. The different color lines represent the different cell proliferation rate (β_{10}). **e.** Time course of the probability of DTP and resistant cells when cell proliferation changes. All parameters are taken in Table A and $\hat{Q}_0$ is set to be 5000 cells.

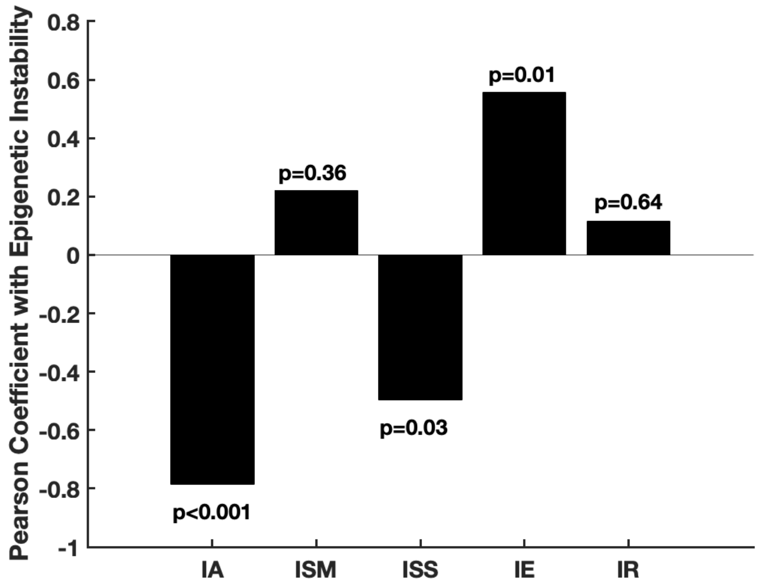

Fig. F. Pearson relationship analysis between epigenetic instability and five subtypes of tumor immune microenvironment in scRNA-seq data. P-values are indicated on the top of each bar. IA：Immune Activation, ISM: Immune Suppressive Myeloid, ISS: Immune Suppressive Stromal, IE: Immune Exclusion, IR: Immune Residence.

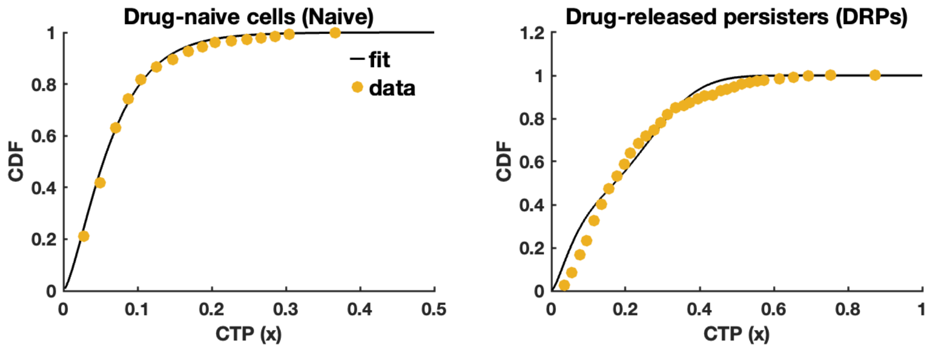

Fig. G. Comparison of CTP distribution between inheritance function $p(x, z)$ and real data from Dataset 1. The black line shows the cumulative probability obtained from default values in Table A. The yellow dots represent real data of CTP distribution from Dataset 1.

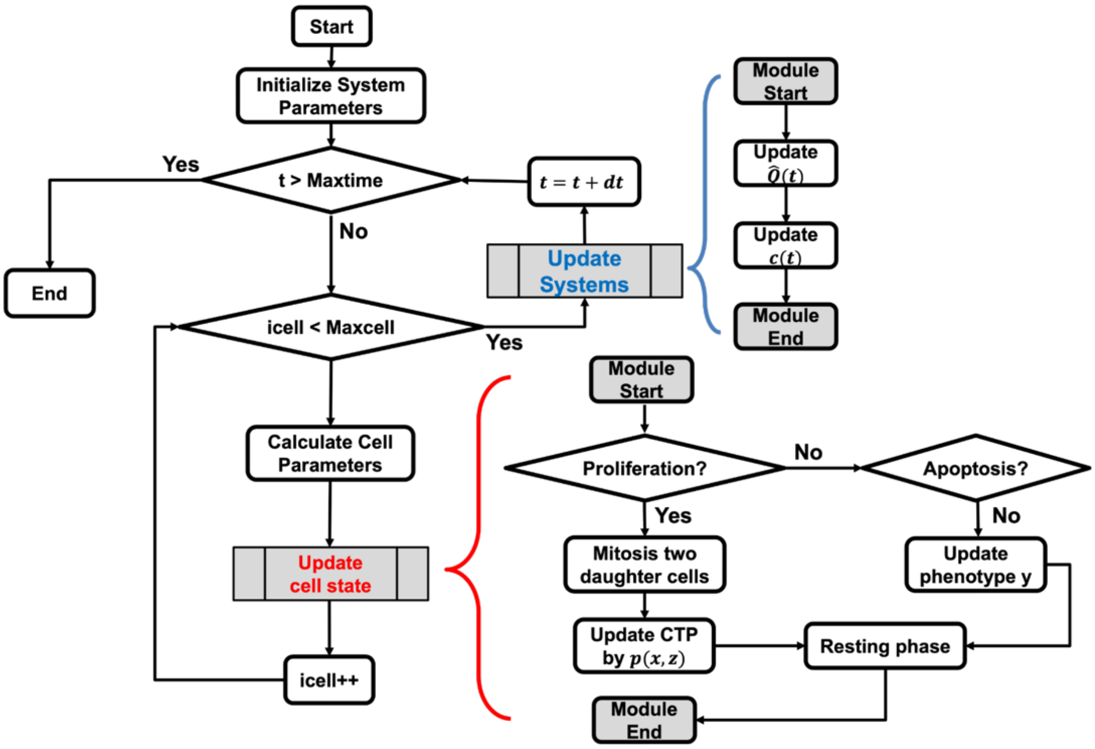

Fig. H. Flow chart of the numerical scheme of the simulation process.

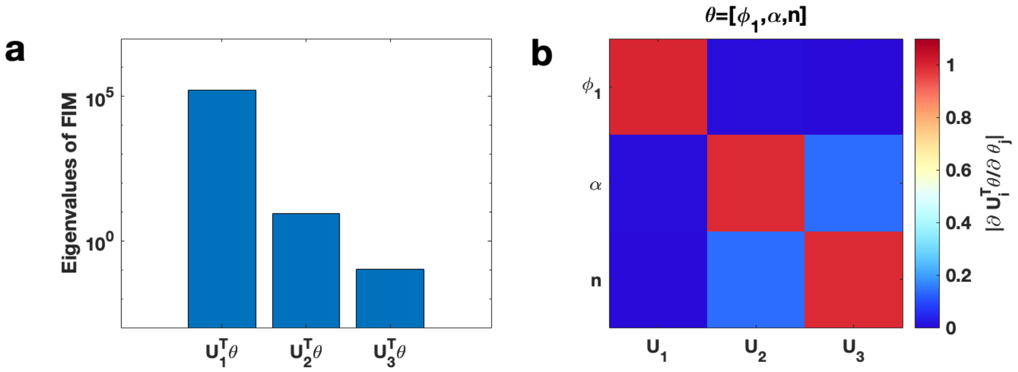

Fig. I. Parameter identifiability of parameters $\phi_1, \alpha,$ and n. a. Eigenvalues of FIM $F(\phi_1, \alpha, n)$ and corresponding eigenvectors $U_i^T\theta$ $(i = 1,2,3)$. b. Coefficient matrix. The colorbar represents the absolute value of coefficient.

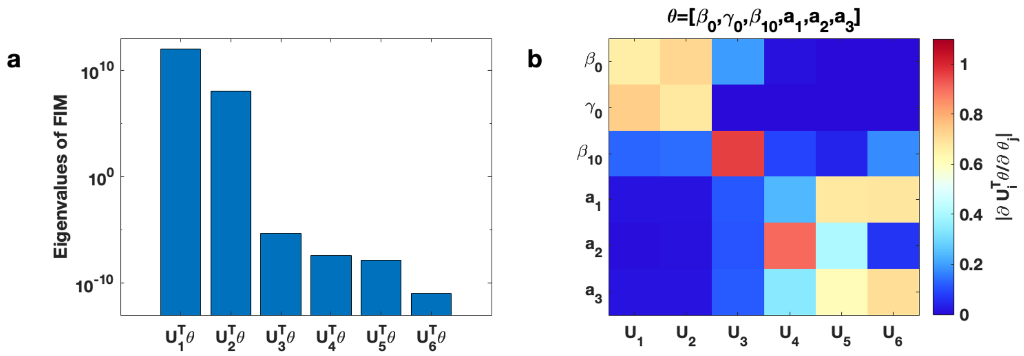

Fig. J. Parameter identifiability of parameters $\beta_0, \gamma_0, \beta_{10}, a_1, a_2$ and a_3. a. Eigenvalues of FIM $F(\beta_0, \gamma_0, \beta_{10}, a_1, a_2, a_3)$ and corresponding eigenvectors $U_i^T \theta$ $(i = 1,2, \ldots, 6)$. b. Coefficient matrix. The color bar represents the absolute value of coefficient.

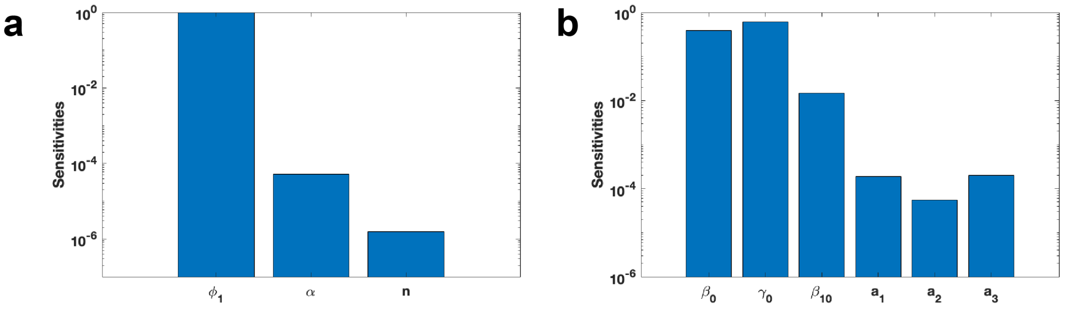

Fig. K. Sensitivity analysis to multiscale parameters. **a**. Estimated parameters ϕ_1, α, n at the epigenetic level. **b**. Estimated parameters $\beta_0, \gamma_0, \beta_{10}, a_1, a_2$ and a_3 at the population level. Sensitivities are denoted as $\mathcal{F}_j$.

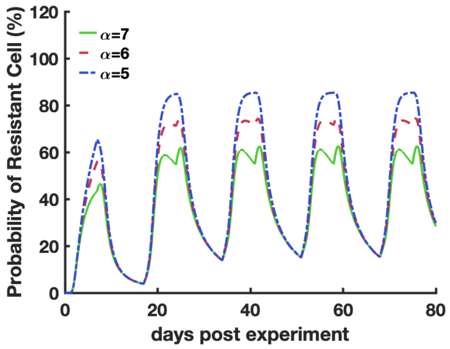

Fig. L. Time course of the probability of resistant cells with different CTP favoring ability α with drug holidays set to 10 days. The different colored lines represent different levels of CTP favoring ability α.

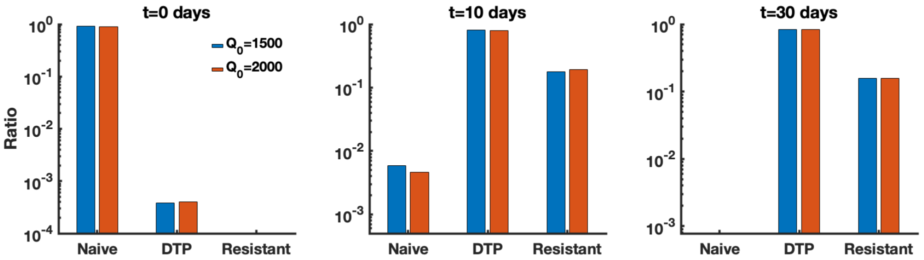

Fig. M. Ratio of three cell types at the different time points when the population size (Q_0) is changed to 2000.

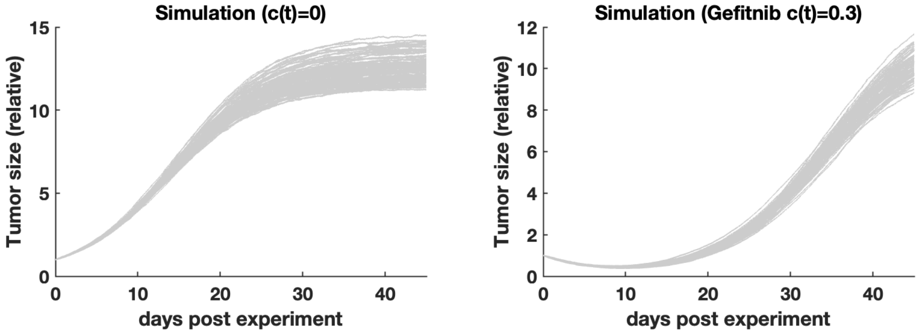

Fig. N. Time course of the tumor size (from 100 independent runs) in both the no-drug and gefitinib treatment cases.

References

1. Jacob Berger A, Gigi E, Kupershmidt L, Meir Z, Gavert N, Zwang Y, et al. IRS1 phosphorylation underlies the non-stochastic probability of cancer cells to persist during EGFR inhibition therapy. Nature Cancer. 2021;2: 1055–1070. doi:10.1038/s43018-021-00261-1
2. Rehman SK, Haynes J, Collignon E, Brown KR, Wang Y, Nixon AML, et al. Colorectal Cancer Cells Enter a Diapause-like DTP State to Survive Chemotherapy. Cell. 2021;184: 226-242.e21. doi:10.1016/j.cell.2020.11.018
3. Miao H, Xia X, Perelson AS, Wu H. On Identifiability of Nonlinear ODE Models and Applications in Viral Dynamics. SIAM Rev. 2011;53: 3–39. doi:10.1137/090757009
4. Komorowski M, Costa MJ, Rand DA, Stumpf MPH. Sensitivity, robustness, and identifiability in stochastic chemical kinetics models. Proceedings of the National Academy of Sciences of the United States of America. 2011;108: 8645–8650. doi:10.1073/pnas.1015814108
5. Gallo L, Frasca M, Latora V, Russo G. Lack of practical identifiability may hamper reliable predictions in COVID-19 epidemic models. Sci Adv. 2022;8: eabg5234. doi:10.1126/sciadv.abg5234
6. Lei J. A general mathematical framework for understanding the behavior of heterogeneous stem cell regeneration. Journal of Theoretical Biology. 2020;492: 110196–110196. doi:10.1016/j.jtbi.2020.110196
7. Lei J, Levin SA, Nie Q. Mathematical model of adult stem cell regeneration with cross-talk between genetic and epigenetic regulation. Proceedings of the National Academy of Sciences of the United States of America. 2014;111: 880–887. doi:10.1073/pnas.1324267111
8. Lei J. Evolutionary dynamics of cancer: From epigenetic regulation to cell population dynamics—mathematical model framework, applications, and open problems. Science China Mathematics. 2020;63: 411–424. doi:10.1007/s11425-019-1629-7
9. Lei J. Systems Biology. Cham: Springer International Publishing; 2021. doi:10.1007/978-3-030-73033-8
10. Guo Y, Nie Q, MacLean AL, Li Y, Lei J, Li S. Multiscale modeling of inflammation-induced tumorigenesis reveals competing oncogenic and oncoprotective roles for inflammation. Cancer Research. 2017;77: 6429–6441. doi:10.1158/0008-5472.CAN-17-1662
11. Lai X, Stiff A, Duggan M, Wesolowski R, Carson WE, Friedman A. Modeling combination therapy for breast cancer with BET and immune checkpoint inhibitors. Proceedings of the National Academy of Sciences of the United States of America. 2018;115: 5534–5539. doi:10.1073/pnas.1721559115
12. Zhang C, Shao C, Jiao X, Bai Y, Li M, Shi H, et al. Individual cell-based modeling of tumor cell plasticity-induced immune escape after CAR-T therapy. Computational and Systems Oncology. 2021;1: 1–13. doi:10.1002/cso2.1029

13. Chisholm RH, Lorenzi T, Lorz A, Larsen AK, De Almeida LN, Escargueil A, et al. Emergence of Drug Tolerance in Cancer Cell Populations: An Evolutionary Outcome of Selection, Nongenetic Instability, and Stress-Induced Adaptation. Cancer Research. 2015;75: 930–939. doi:10.1158/0008-5472.CAN-14-2103
14. Kessler DA, Levine H. Phenomenological Approach to Cancer Cell Persistence. Physical Review Letters. 2022;129: 108101–108101. doi:10.1103/physrevlett.129.108101
15. Xue R, Zhang Q, Cao Q, Kong R, Xiang X, Liu H, et al. Liver tumour immune microenvironment subtypes and neutrophil heterogeneity. Nature. 2022;612: 141–147. doi:10.1038/s41586-022-05400-x
